# Compound A398, a Novel Podophyllotoxin Analogue: Cytotoxicity and Induction of Apoptosis in Human Leukemia Cells

**DOI:** 10.1371/journal.pone.0107404

**Published:** 2014-09-15

**Authors:** Alethéia L. Silveira, Glaúcia V. Faheina-Martins, Raquel C. Maia, Demetrius A. M. Araújo

**Affiliations:** 1 Departamento de Biotecnologia, Universidade Federal da Paraíba, João Pessoa, Paraíba, Brasil; 2 Departamento de Biologia Molecular, Universidade Federal da Paraíba, João Pessoa, Paraíba, Brasil; 3 Laboratório de Hemato-Oncologia Celular e Molecular, Instituto Nacional de Câncer (INCA), Rio de Janeiro, Rio de Janeiro, Brasil; Wayne State University, United States of America

## Abstract

Despite advances in oncology research, cancer is one of the leading causes of death worldwide. Thus, there is a demand for the development of more selective and effective antitumor agents. This study showed that A398, a novel podophyllotoxin analogue, was cytotoxic to the HT-29, MCF-7, MOLT-4 and HL-60 tumor cell lines, being less active in human peripheral blood mononuclear cells and normal cell lines FGH and IEC-6. Tests using the HepG2 lineage indicated that its metabolites do not contribute to its cytotoxicity. In the HL-60 cells, A398 induced apoptosis in a time and concentration-dependent manner, promoting mitochondrial depolarization, inhibition of Bcl-2, phosphatidylserine exposure, activation of caspases -8, -9 and -3, and DNA fragmentation. The production of reactive oxygen species does not seem to be a crucial event for the apoptotic process. Pretreatment with specific inhibitors of kinases ERK1/2, JNK and p38 resulted in an increased percentage of death induced by A398. These results indicate that the compound induced apoptosis through activation of intrinsic and extrinsic death pathways with the mechanism involving the inhibition of the MAPKs and Bcl-2. Taken together, our findings suggest that A398 has an anticancer potential, proving itself to be a candidate for preclinical studies.

## Introduction

Existing schemes of therapeutic intervention for cancer treatment depend on the ability to induce tumor cell death by apoptosis [1], [2]. This consists of a highly regulated, energy-dependent cell program, which plays an important role in many biological processes such as growth, proliferation, differentiation, immunity, removal of unwanted cells and the maintenance of homeostasis of the tissues [3], [4], [5].

The cascade of the apoptosis process may be mediated through two main pathways: the extrinsic and the intrinsic [6].The extrinsic pathway is initiated by the interaction of death ligands to their respective receptors present on the surface of the plasma membrane and results in activation of caspases -8 and -10 [7], [8]. The intrinsic or mitochondrial pathway is triggered by a variety of stimuli such as ultraviolet light, reactive oxygen species, the action of cytotoxic agents and DNA damage [9], [10]. The changes that occur in the inner mitochondrial membrane promote the release of several pro-apoptotic proteins into the cytosol, such as cytochrome C, Smac/DIABLO (Second mitochondrial activator of caspases/direct IAP binding protein with low PI) and endonuclease G. After being released from the mitochondria, cytochrome C binds to the adapter protein Apaf-1 (Apoptotic protease activating factor) to form the apoptosome, with activation of caspase -9 [11], [12], [13].

The extrinsic and intrinsic pathways converge at the phase of apoptosis execution, which is mediated mainly by effector caspases -3, -6 and -7 [14], [15]. The cleavage of specific cellular substrates results in biochemical and morphological changes, such as: cell shrinkage, membrane blebbing, phosphatidylserine exposure, chromatin condensation and nuclear fragmentation [16], [17]. Due to the physiological and pathological importance of cell death by apoptosis, this process is strictly regulated by several proteins, such as p53, members of the Bcl-2 family (B-cell lymphoma protein 2) and MAPKs (Mitogen-activated protein kinases) [18], [19], [20].

Natural products have been a rich source of agents of value to medicine. More than half of currently available drugs are natural compounds or are related to them, and in the case of cancer this proportion surpasses 60% [21], [22]. Podophyllotoxin is a natural cyclolignan isolated from various plant species within the *Podophyllum* family. The cytotoxic effects of podophyllotoxin in different types of tumors have been attributed to its ability to bind to tubulin during mitosis and thus inhibit microtubule assembly. Unfortunately, its high toxicity has limited its application as a chemotherapeutic [23], [24]. Continuous efforts towards the synthesis of its analogues led to the discovery of new anticancer drugs. For example its semisynthetic derivatives, etoposide and teniposide are currently used in the clinic for the treatment of a variety of malignancies. Due to the drug resistance developed by cancer cells as well as side effects associated with the use of these agents in clinic, the search for new effective anticancer analogues of podophyllotoxin remains an intense area of research [25], [26], [27]. In this study we evaluated the anticancer potential of a new podophyllotoxin analogue called (2S,6S,7R)-4-(4-fluoro-3-nitrophenyl)-7-methyl-12,14-dioxo-4-azatetracycle [7.7.0.02,6.011,15] hexadec-1(16),9,11(15)-triene-2,5-dione, which was coded as A398 (patent pending number PI1102759-2, deposited at the National Institute of Industrial Property, Brazil).

## Materials and Methods

### Ethics Statement

This study was approved by the Institutional Ethical Committee of Lauro Wanderley Hospital from Federal University of Paraíba, number 05878712.7.0000.5183. We used Human peripheral blood mononuclear cells (PBMC), which were isolated from samples of peripheral blood from healthy non-smoking donors who had not taken any medication for at least 15 days before donation, aged 18–30 years. As described in such approved document, these blood samples were provided by the Blood Center of Paraíba (Hemocentro, João Pessoa, Paraíba, Brazil) with the permission of blood donors and we also mention that we just used the blood samples that would be discarded by the Hemocentro.

### Chemicals and antibodies

Propidium iodide (PI), 3-(4,5-dimethyl-thiazol-2-yl)-2,5-diphenyl-terazolium bromide (MTT), SB 203580, SP 600125, PD 98059, etoposide, tetramethyl rhodamine methyl ester (TMRM), phytohemagglutinine and carbonyl cyanide m-chlorophenyl hydrazone (CCCP) were purchased from Sigma (St. Louis, MO, USA). Alexa Fluor 488 and 2′,7′-dichlorodihydrofluorescein diacetate (H_2_DCFH-DA) were obtained from Invitrogen (Carlsbad, CA, USA). Anti-Bcl-2 and β-actin were purchased from Santa Cruz Biotechology (Santa Cruz, CA, USA). All organic solvents and other chemicals were of analytical grade or complied with the standards needed for cell culture experiments.

### Cell cultures

HL-60 (human acute promyelocytic leukemia), MOLT-4 (human acute lymphoblastic leukemia), MCF-7 (human breast cancer), HT-29 (human colon cancer), HepG2 (human hepatoma), FGH (human gingival fibroblasts) and IEC-6 (rat intestinal epithelial) cell lines were obtained from the Rio de Janeiro Cell Bank (Brazil). Cells were culture in RPMI 1640 medium (for the HL-60, MOLT-4, MCF-7 and IEC-6 cells) or DMEM medium (for the HT-29, HepG2 and FGH lineages), supplemented with 10% FBS and antibiotics (100 UI/mL of penicillin and 100 µg/mL of streptomycin). Alls cells were maintained in a 5% CO_2_ humidified incubator at 37°C.

PBMC cells were isolated from heparin-anticoagulated blood of healthy volunteers by centrifugation with Ficoll-Paque Plus (Amersham Biosciences). PBMCs were also stimulated with phytohemagglutinine (PHA, 2 µg/ml) for 24 h before experimental treatment and were culture in RPMI 1640 medium.

### Cytotoxicity assay

Cell viability was evaluated by measuring the mitochondrial-dependent reduction of MTT to a colored blue formazan. Briefly, cells was added to 96-well plates and incubated with increasing concentrations of compound A398 and etoposide for 24 hours. After the time exposure, MTT (5 mg/mL) was added and the plates were incubated at 37°C for 4 h. Formazan crystals were solubilized by the addition of 10% SDS in 0.01 N HCl overnight and the absorbance was measured at 570 nm on a microplate reader (Biotek Instruments EL800, USA). The cytotoxicity was expressed as an IC_50_ (concentration that inhibits 50% of the growth of cells), which was determined from the concentration-response curve.

### Evaluation of apoptosis

The extent of apoptosis was quantified using annexinV/PI dual staining. HL-60 cells (5×10^5^ cells/well) were exposed to compound A398 (4, 6 or 8 µM) or etoposide (5 µM) for 1 h, 3 h and 6 h. After the treatment periods, the cells were centrifuged (1.500 rpm, 5 minutes) and stained with 1 µl of annexin V-alexa Fluor 488 and 2 µl of 50 µg/ml PI in 100 µl binding buffer [10 mM HEPES (pH 7.4), 140 mM NaOH and 2.5 mM CaCl_2_] for 15 min at room temperature in the dark. Quantification of apoptotic cells was performed by flow cytometry using a FASC Calibur (BD Biosciences, USA).

### Determination of mitochondrial membrane potential (ΔΨm)

Mitochondrial membrane potential was measured using TMRM, a cationic fluorochrome that rapidly accumulates in the mitochondria of living cells. HL-60 cells (5×10^5^ cells/well) were treated with A398 (4, 6 or 8 µM) or with 5 µM of etoposide. As a positive control, cells were treated with 50 µM of the protonophore CCCP for 15 min. After 1 h, 3 h, and 6 h of exposure, the samples were centrifuged (1.500 rpm, 5 minutes) and stained with 150 nM of TMRM for 20 minutes in the dark at 37°C. The cells were then washed with PBS and analyzed by flow cytometry.

### Estimation of caspases -8, -9, and -3 activity

HL-60 cells (2×10^6^ cells/well) were incubated with A398 (6 µM) or etoposide (5 µM) for 1 h, 3 h, 6 h and 12 h. The activities of the caspases were carried out using colorimetric protease assay (Invitrogen, USA) following the manufacturer's protocol. Each kit contains a specific substrate: IETD (Ile-Glu-Thr-Asp), LEHD (Leu-Glu-His-Asp) and Ac-DEVD (acetyl-Asp-Glu-Val-Asp) for caspases -8, -9 and -3, respectively. Such substrates are labeled to the chromophore *p*-nitroanilide (*p*NA), which is released when they are cleaved by activated caspases and measured at 405 nm in a spectrophotometer (Biotek Instruments EL800, USA).

### Cell cycle analysis and DNA content

Propidium iodide is a commonly used dye to quantitatively assess DNA content in cell cycle analysis. HL-60 cells (5×10^5^ cells/well) were incubated with A398 (2, 4 or 6 µM) or etoposide (5 µM) for 1 h, 3 h and 6 h. The samples were centrifuged (1.500 rpm, 5 minutes) and resuspended in a hypotonic solution containing 0.1% sodium citrate, 0.1% triton X-100, RNase (10 µg/mL) and PI (50 µg/mL). The mixture was incubated in the dark at 4°C for 30 min and was then acquired in a FACS Calibur (BD Biosciences, USA).

### Analysis of DNA fragmentation

DNA fragmentation was qualitatively analyzed by gel electrophoresis. HL-60 cells (2×10^6^ cells/well) were treated with A398 (6 µM) or etoposide (5 µM). After 3 h, 6 h and 12 h, the cells were centrifuged (1,500 rpm, 5 minutes), washed with PBS and lysed with 250 µL of lysis buffer (10 mM EDTA, 50 mM Tris-HCL, 0.5% SDS) for 15 min at 55°C. Then proteinase K was added (500 µg/mL) and the samples were incubated for 1 h at 55°C. RNAse (1 µg/µL); was then added and the incubation continued for 90 more minutes at 55°C. The DNA was extracted with 250 µL of a solution containing phenol, chloroform and isoamyl alcohol (25∶24∶1) and precipitated with 0.1 volume of NaCl (2 M) and 2.5 volume of absolute ethanol. The precipitate was centrifuged at 12.000 rpm for 10 minutes and dissolved in a solution of Tris (10 mM) and EDTA (1 mM). The samples were separated on agarose gel (1.5%), stained with ethidium bromide (0.5 µg/mL), visualized under ultraviolet light and photographed.

### Participation of MAPKs

To verify the involvement of MAPKs in the apoptosis process, HL-60 cells (5×10^5^ cells/well) were pretreated with p38 inhibitor-SB 203580 (5 µM), JNK inhibitor-SP 600125 (10 µM) or ERK inhibitor-PD 98059 (10 µM) for 1 h and then incubated with derivative A398 (6 µM) for 6 h. The induction of apoptosis was determined by analyzing the sub-G_1_ DNA damage using a FASC Calibur.

### Measurement of intracellular oxygen species (ROS)

The intracellular ROS was estimated by fluorescent probe H_2_-DCF-DA. HL-60 cells (5×10^5^ cells/well) were treated with A398 (4, 6 or 8 µM) or with 5 µM of etoposide. Hydrogen peroxide (50 µM) was used as a positive control. After 1 h, 3 h and 6 h of exposure, the samples were centrifuged (1.500 rpm, 5 min), washed with PBS at 37°C and labeled with 10 µM of H_2_-DCFH-DA for 30 min., in the dark, at 37°C. The cells were then washed with PBS and analyzed by flow cytometry.

### Western blot analysis

HL-60 cells (2×10^6^ cells/mL) were treated with 6 µM of compound A398 for 1 h, 3 h, 6 h and 12 h. Protein concentration was determined using Lowry assay and samples containing 50 µg of proteins were loaded and separated by sodium dodecyl sulphate-polyacrylaminde gel electrophoresis (10%). Protein bands were transferred to nitrocellulose membranes (Hybond-ECL - Amersham Biosciences, USA), blocked with 5% fat free dry milk and then incubated with specified primary antibodies (anti-Bcl2). After washing and incubation with an appropriate horseradish peroxidase-conjugated secondary antibody, the antigen-antibody complexes were visualized by enhanced chemiluminescence (ECL, Amersham Biosciences) using the manufacturer's protocol.

### Statistical analysis

Data from the experiments were obtained from at least three independent tests performed in triplicate. Data were expressed as mean ± standard error of the mean (SE) and analyzed using Analysis of Variance (ANOVA) followed by the Newman-Keuls test, and were considered significant at p<0.05. The statistical software used was GraphPad Prism (GraphPad Software Incorporated, USA). In the cytometry experiments, 10.000 events were collected for each sample and data were analyzed using Summit software (Dako, USA).

## Results

### A398 is cytotoxic in human tumor lineages

**Figure 1:**
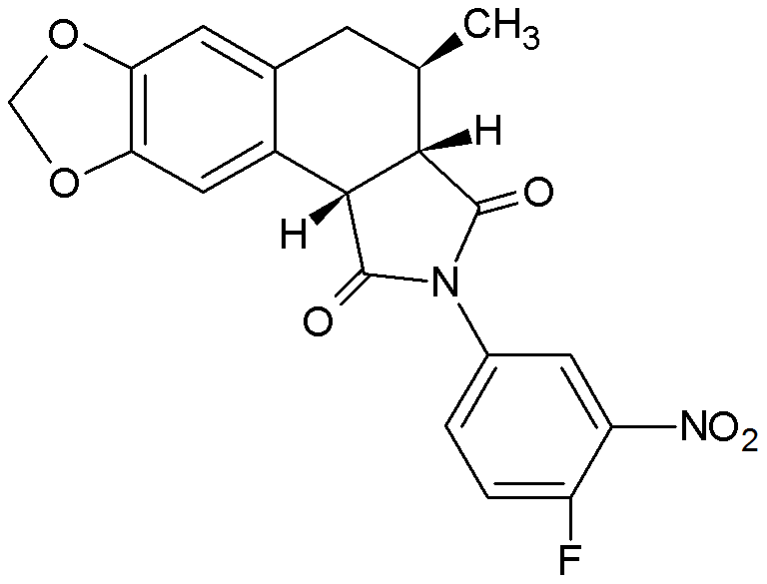
Chemical structure of A398.

The cytotoxic potential of A398 (Figure 1) was evaluated on several human tumor cell lines as well as normal FGH and IEC-6 cell lines and PBMC cells. As shown in table 1, A398 displayed broad and potent cytotoxicity on the tested human cancer cell lines but relatively minor cytotoxicity to normal cells. HL-60 cells were relatively more sensitive to compound A398 and were therefore selected for further analysis.

**Table 1:** Cytotoxicity of A398 and etoposide against several tumor cell lines (MCF-7, HT-29, MOLT-4 and HL-60) and normal cells (FGH, IEC-6 and PBMC).

Compound	IC_50_ (µM)						
	MCF-7	HT-29	MOLT-4	HL-60	FGH	PBMC	IEC-6
A398	41.4±1.6	37.9±2.3	7.3±1.8	6.3±0.8	241.2±15.8	75.3±2.6	127.4±3.8
Etoposide	42.3±8.1	72.9±8.6	0.8±0.3	2.2±1.4	183.1±18.5	102.6±2.4	-

Cells were treated with increasing concentrations of the substances for 24 h and cell viability was evaluated by MTT assay. Each IC_50_ value represented mean ± SE of three independent experiments.

### A398 metabolites do not seem to contribute to its cytotoxic effect

To examine whether derivative A398 produces more toxic metabolites and to determine which enzymes are involved in their metabolism, the cytotoxicity of this compound was evaluated on the HepG2 hepatocytes lineage, using the MTT assay, with the absence and presence of dexamethasone (CYP3A inductor), ketoconazole (CYP3A inhibitor) or borneol (an inhibitor of UDP-glucuronosyltransferases - UGTs). Co-incubation with ketoconazole or with borneol promoted an increase in the cytotoxic effect of A398 while pretreatment with dexamethasone decreased the cytotoxicity of this compound. These results suggest that reactions involving the enzymes CYP3A and UGTs favor the detoxification of the compound (Table 2).

**Table 2:** Effect of inhibitors and inducers of cytochrome P450 isoenzymes on the cytotoxicity of A398.

Compound	IC_50_ (µM)
A398	226.6±11.8
A398 + Dexamethasone	>250
A398 + ketoconazole	133.3±13.5
A398 + Borneol	170.1±7.3

Cell viability was assessed by the MTT method after 24 h of exposure. The treatment of HepG2 cells with A398 was done in the absence or presence of dexamethasone (inductor CYP3A), ketoconazole (inhibitor CYP3A) or borneol (inhibitor UGTs). In the latter condition, cells were pretreated with 10 µM of dexamethasone (for 24 h) or co-incubated with 15 µM ketoconazole or with 500 µM borneol. Each IC50 value represented mean ± SE of three independent experiments.

### Apoptosis induced by A398 involves changes in the mitochondrial transmembrane potential (ΔΨm)

**Figure 2:**
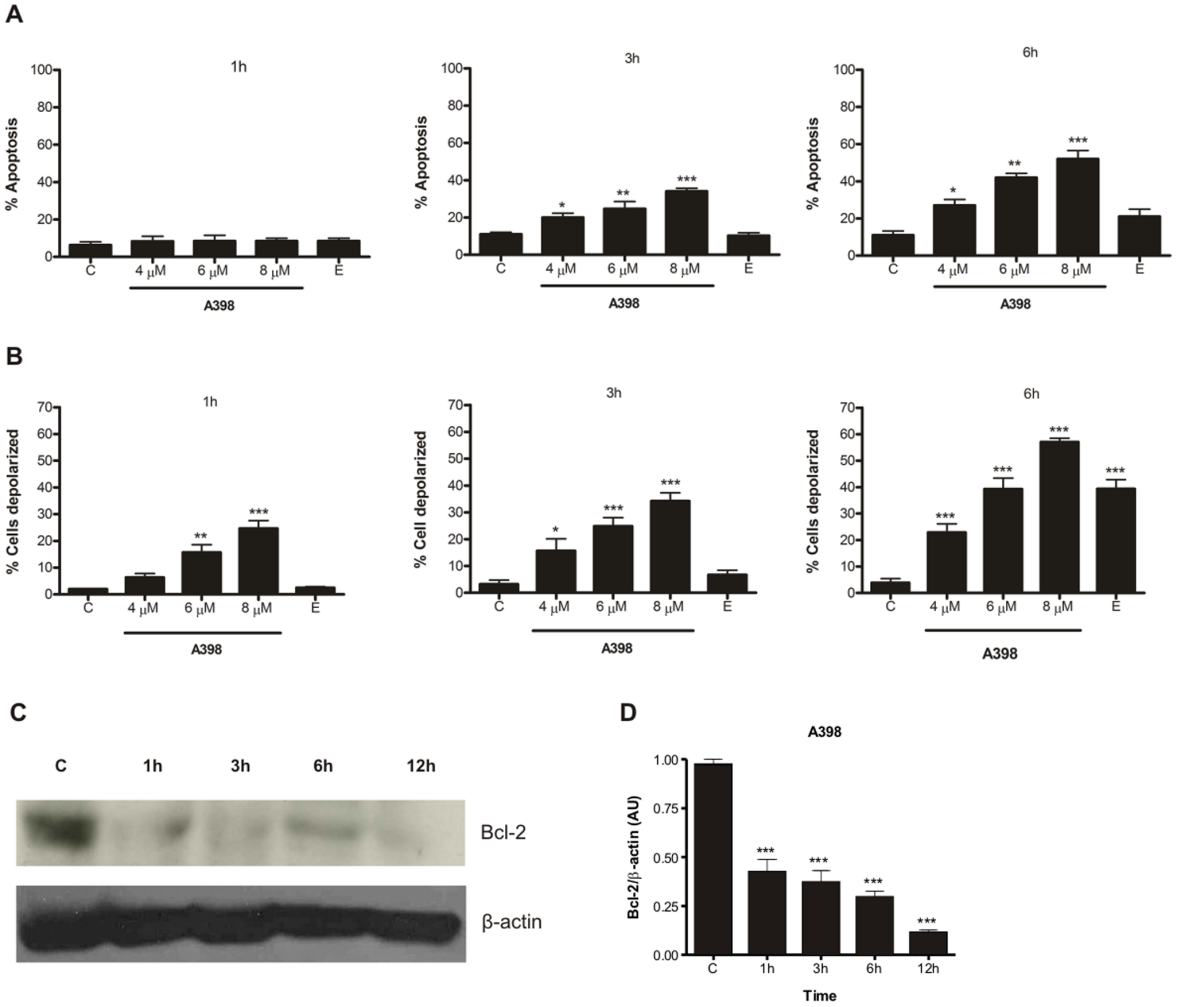
A398 induces apoptosis in HL-60 cell line. Cells were treated with A398 (4, 6 and 8 µM) or etoposide (5 µM) for 1 h, 3 h or 6 h and stained with annexin V-alexa fluor 488 and PI or with TMRM. The rate of apoptosis (A) and the percentage of depolarized cells (B) were quantified by flow cytometry. (C) Decreased expression of Bcl-2 in HL- 60 lineage. Cells were treated with 6 µM of A398 for 1 h, 3 h, 6 h and 12 h. The bands were revealed on nitrocellulose membrane after staining with secondary antibody labeled with peroxidase. β-actin was used as a loading control. C: control. (D) Bar plot showing arbitrary unit (AU) obtained from densitometry measure displayed in (C). Data are expressed as mean ± SE of three independent experiments. *p<0.05, **p<0.01 and ***p<0.001 compared with the control by ANOVA.

In order to determine whether A398 has an apoptosis-inducing activity on HL-60 cell line, dual staining with annexin V/PI was performed. Death promoted by A398 was time and concentration-dependent. The percentage of apoptotic cells significantly increased with 3 h of treatment and approximately 49.10% of the population was undergoing apoptosis after 6 h. Etoposide did not induce apoptosis until 6 h after the treatment (Figure 2A and Figure S1 in File S1).

To examine whether there is mitochondrial pathway involvement in apoptosis induced by A398, changes in ΔΨm were evaluated using the fluorochrome TMRM. The results showed that A398 promoted an increase in mitochondrial membrane depolarization in a time and concentration-dependent manner. A significant increase in the percentage of depolarized cells was detected with 1 h of treatment and reached 57.11% after 6 h. Alterations in ΔΨm induced by etoposide were detected at 6 h of incubation (Figure 2B and Figure S2 in File S1). Given that Bcl-2 is an anti-apoptotic protein that regulates mitochondrial outer membrane permeabilization, the effect of A398 on the expression of this protein was investigated using western blot. As shown in Figures 2C and 2D, the compound almost completely inhibited the expression of Bcl-2 at 1 h of incubation, which suggests that this protein is involved in regulating the intrinsic pathway of death.

### Cell death promoted by A398 is mediated by activation of caspases

**Figure 3:**
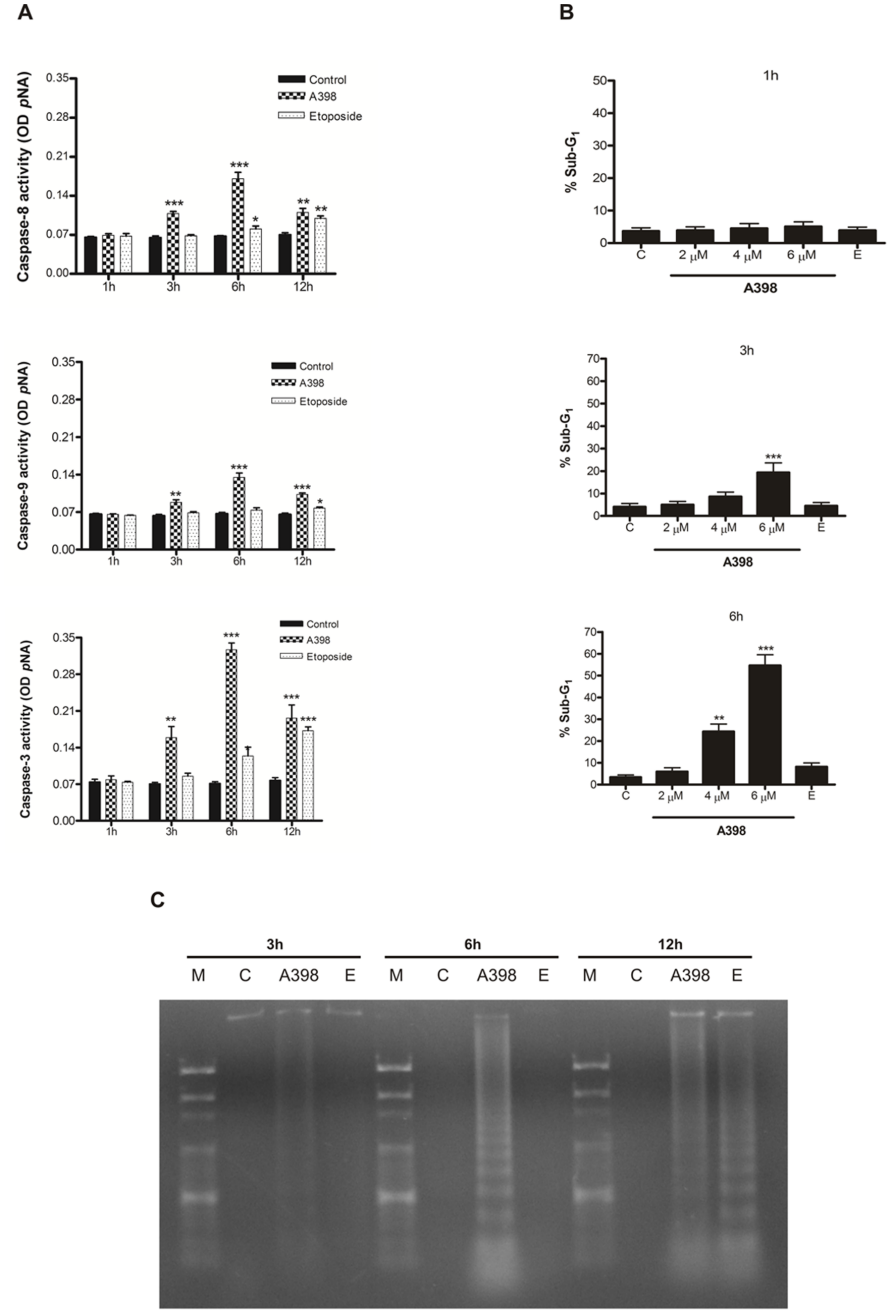
Activation of caspases and induction of DNA fragmentation by A98 in HL-60 cells. (A) Analysis of caspase activation by A398 in HL-60 lineage. Cells were treated with 6 µM of A398 or with 5 µM of etoposide for 1 h, 3 h, 6 h and 12 h. The level of activity of each caspase was expressed in relation to the optical density (OD) of the chromophore *p*NA. (B) A398 induces an increase in the sub-G_1_ fraction in HL-60 lineage. Cells were treated with A398 (2, 4 and 6 µM) or etoposide (5 µM) for 1 h, 3 h or 6 h. After incubation, cells were stained with PI and analyzed by flow cytometry. (C) A398 promoted internucleosomal DNA fragmentation in HL-60 lineage. Cells were treated with 6 µM of A398 or 5 µM of etoposide for 3 h, 6 h and 12 h. DNA was extracted, separated on agarose gel and visualized with UV light. C: control; M: marker; E: etoposide. Data are expressed as mean ± SE of three independent experiments. *p<0.05, **p<0.01 and ***p<0.001 compared with the control by ANOVA.

Because of the importance of caspases as executors of the apoptotic process, the participation of caspases -8, -9 and -3 was evaluated using specific fluorogenic substrates. As shown in Figure 3A, A398 promoted the significant activation of caspases -8, -9 and -3 after 3 hours of treatment and the maximum activity was reached at 6 hours of exposure. Etoposide induced a significant activation of caspases -8 and -3 after 6 h of treatment, while an increase in the activity of caspase -9 was only observed at 12 h.

### Effects of A398 on the cell cycle and DNA content

To assess whether the induction of apoptosis by A398 is associated with changes in the distribution profile of cells in some stages of the cell cycle, we used the dye propidium iodide. In all times tested, A398 did not promote cell cycle arrest. However, the appearance of cells with hypodiploid content (sub-G_1_), a characteristic of apoptotic cells with condensed nuclei and fragmented DNA, was time and concentration-dependent. The results show that the cytotoxic effect of A398 is not phase-specific, unlike etoposide, which promoted an accumulation of cells in S phase after 3 h and 6 h of treatment (Figure 3B and Figure S3 in File S1).

### A398 induces damage to DNA

The degradation of DNA into fragments of 180–200 base pairs is a striking characteristic of death by apoptosis. Figure 3C shows that A398 promoted typical internucleosomal DNA fragmentation resulting from the induction of apoptosis or “ladder” formation all time points tested. The fluorescence of the bands was weak in the period of three hours, where maximum intensity was reached at a period of 6 h, with a reduction after 12 hours of exposure. Etoposide, when used in a concentration similar to that of compound A398, only induced DNA fragmentation in the period of 12 hours of treatment.

### Effect of MAPKs inhibitors on apoptosis promoted by A398

**Figure 4:**
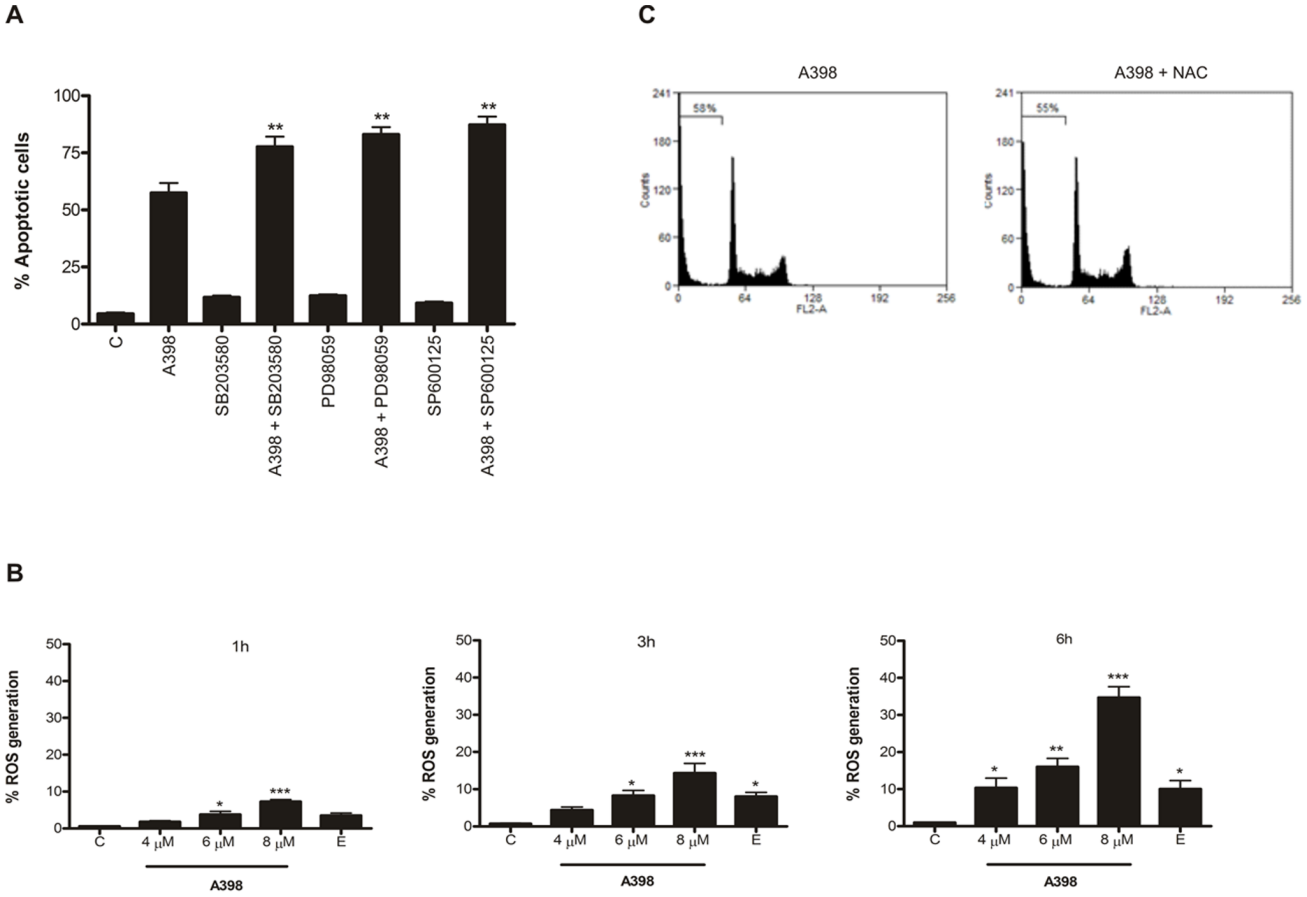
A398 inhibits the activation of MAPk and induces ROS generation in the HL-60 cells. (A) Effect of MAPKs inhibitors on apoptosis promoted by A398 in HL-60 lineage. Cells were pretreated with ERK inhibitor-PD 98059 (10 µM), JNK inhibitor-SP 600125 (10 µM), or p38 inhibitor-SB 203580 (5 µM) for 1 h and incubated with A398 (6 µM) during 6 h. Cells were stained with PI and analyzed by flow cytometry for sub-G_1_ DNA damage. Data are expressed as mean ± SE of three independent experiments. **p<0.01, significantly different from derivative A398 treatment alone. (B) Evaluation of A398-induced ROS generation in HL-60 lineage. Cells were treated with A398 (4, 6 and 8 µM) or etoposide (5 µM) for 1 h, 3 h or 6 h, labeled with H_2_- DCFH-DA and ROS production was quantified by flow cytometry. (D) Effect of antioxidant NAC on the induction of death of A398 in HL-60 lineage. Cells were pretreated with NAC and exposed to the compound (6 µM) for 6 h. After incubation, cells were stained with PI and analyzed by flow cytometry for sub-G_1_ DNA damage. Data are expressed as mean ± SE of three independent experiments. *p<0.05, **p<0.01 and ***p<0.001 compared with the control by ANOVA.

MAPK pathways can mediate signals that either promote or suppress the growth of malignant hematopoietic cells. In view of evidence that p38, ERK, and JNK play a critical role in cell fate, the effect of A398 on these kinases was examined. Pretreatment with the inhibitors SB203580, PD98059 and SP600125 resulted in an increase in the percentage of death induced by A398 of 10.5, 14.4 and 22.8%, respectively (Figure 4A). This result suggests that A398 inhibits kinases p38, ERK1/2 and JNK and this effect certainly contributes at least in part, to the sensitivity of these leukemia cells to follow the apoptotic pathway.

### Reactive oxygen species are not crucial for A398 to induce death

Given that oxidative stress may participate in the process of apoptosis, we assessed whether derivative A398 promotes the generation of ROS using the probe H_2_-DCFH-DA. Derivative A398 elevated ROS production in a time and concentration-dependent manner and the percentage of cells generating ROS reached 35.57% after 6 h of treatment (Figure 4B and Figure S4 in File S1). To determine whether reactive oxygen species may be involved in the process of apoptosis initiation, cells were pretreated with the antioxidant N-acetyl-cysteine (NAC) for 1 hour and subsequently exposed to A398 (6 µM) for 6 h. The induction of death was assessed by percentage of the sub-G_1_ fraction. Figure 4C shows that pretreatment with NAC did not alter the percentage of cells in sub-G_1_, suggesting that the generation of ROS appears not to be crucial for the induction of apoptosis by A398.

## Discussion

Within the sphere of cancer, an important number of drugs currently commercialized were obtained from natural sources or their derivatives. Some of the compounds with chemotherapeutic activity, such as podophyllotoxin, serve as prototypes for the synthesis of new molecules with better pharmacological activity [28], [23], [22]. The present study showed that A398, a new podophyllotoxin analogue, was cytotoxic on different types of tumor cells and least active against normal cells.

In the early stages of cytotoxicity evaluation of a new substance, the use of hepatic cell lineages serves to detect the presence of more toxic metabolites and to assess which enzymes are involved in the metabolism of the substance being studied [29], [30]. In this context, the cytotoxicity of A398 was assessed in the HepG2 lineage. The results suggest that the metabolites produced by A398 do not contribute to its cytotoxic activity and that metabolic reactions involving the enzymes CYP3A (phase I) and UGTs (phase II) favor the detoxification of the compound.

The suppression of apoptotic mechanisms is involved both in the process of tumorigenesis and maintenance of the malignant phenotype as in the development of drug resistance [31], [12]. Therefore, the development of compounds that induce apoptosis is one of the therapeutic strategies in the field of oncology [6], [32], [4]. Our results showed that A398 promoted concentration- and time-dependent apoptosis in leukemia cell line HL-60.

The mitochondria integrates numerous signals of cell survival and death, thus exerting a decisive control over the various biochemical cascades that lead to apoptosis [33], [34], [13]. The BCL-2 family of proteins constitutes a critical control point in apoptosis residing immediately upstream of irreversible cellular damage, where family members control the release of apoptogenic factors from mitochondria [35], [19]. Caspases are a family of cystine proteases which serve critical roles in initiation and execution of apoptosis [15], [36]. Our studies clearly demonstrated that A398 promoted dissipation of mitochondrial transmembrane, inhibited the expression of anti-apoptotic Bcl-2 protein and induced activation of caspases -8, -9 and -3 in HL-60 cells, confirming the involvement of both intrinsic and extrinsic apoptotic pathways in apoptosis induced by A398.

In response to several types of cellular stress, cells may promote cell cycle arrest so as to prevent the progression of mutated and damaged cells [37]. Compound A398 exerted its cytotoxic effect on the HL-60 lineage without causing cell cycle arrest, with an increase in the sub-G_1_ fraction, which shows induction of apoptosis. It is possible that the damage caused by this drug is so intense that there is no time for cell cycle arrest in an attempt to repair DNA, occurring rapidly an activation of apoptotic machinery [38], [39]. Indeed, the results showed that A398 exerted its apoptotic effects early, much faster than etoposide in concentrations similar to this. Although cells are killed by etoposide in any moment of the cell cycle, several data indicate a higher sensitivity from mid-S to G_2_ phase [58]. Our studies demonstrated that etoposide promote arrest in the S-phase in HL-60, showing that A398 had a distinct mechanism of action compared to etoposide.

The degradation of DNA in a specific pattern of fragmentation is a characteristic of apoptosis, occurring due to activation of caspases -3 and endonucleases [40], [41]. The electrophoresis in agarose gel showed that A398 promoted internucleosomal DNA fragmentation. Together with the other results, we found that the effector phase of the apoptotic process triggered by A398 starts at a time period of 3 hours, reaching a maximum induction of death in 6 hours of treatment, with a diminished effect from 12 hours treatment.

The three major mitogen-activated protein kinases (MAPKs) p38, JNK (c-Jun NH2-terminal kinases), and ERK (extracellular signal-regulated kinases) are signal transducers involved in a broad range of cell functions including survival, apoptosis, and cell differentiation [42], [43]. Mutations leading to constitutive activation of the pathway Ras/Raf/MEK/ERK are highly prevalent in hematological cancers and mediates mitogenic and anti-apoptotic signals in leukemia cells [44], [45]. Several studies suggest that p38 and JNK play pivotal roles in the leukemogenesis and may contribute to increased resistance to chemotherapeutics [46], [47]. A398 appear to inhibit the activation of ERK1/2, p38 and JNK in HL-60 cells and this effect can contribute to the sensitivity of leukemia cells to the apoptotic effects of compound.

Reactive oxygen species (ROS) may play an important role in intracellular signaling cascades that trigger the initiation of apoptotic death [48], [49]. The results showed that treatment with A398 promoted an increase in ROS. However, ROS does not seem to play a critical role in the initiation of apoptosis induced by A398, since the pretreatment of cells with the antioxidant NAC did not alter the percentage of death induced by this compound. Another evidence is that A398 did not promote the activation of the JNK and p38 pathways, which consist of the main mechanism by which ROS mediates the pro-apoptotic signaling [50], [51]. Since the mitochondria becomes a source of generation of reactive oxygen species during apoptosis, we suggest that ROS production occurs after the dissipation of ΔΨm, and may be involved in the amplification of death by apoptosis through the intrinsic pathway, since ROS promotes the depolarization of mitochondria [48], [50].

The tumor suppressor protein p53 plays a critical role in regulating cell death, using various mechanisms to promote apoptosis [52], [53]. Since the HL-60 cells are p53-deficient [54], [55], A398 operates independently of the activation of this protein. So, this compound may also be effective in other tumor cells that have no functional p53, which is very important since the mutation and inactivation of this protein occurs in more than half of all types of cancer, and is correlated with the degree of aggressiveness of some tumors [56], [57].

**Figure 5:**
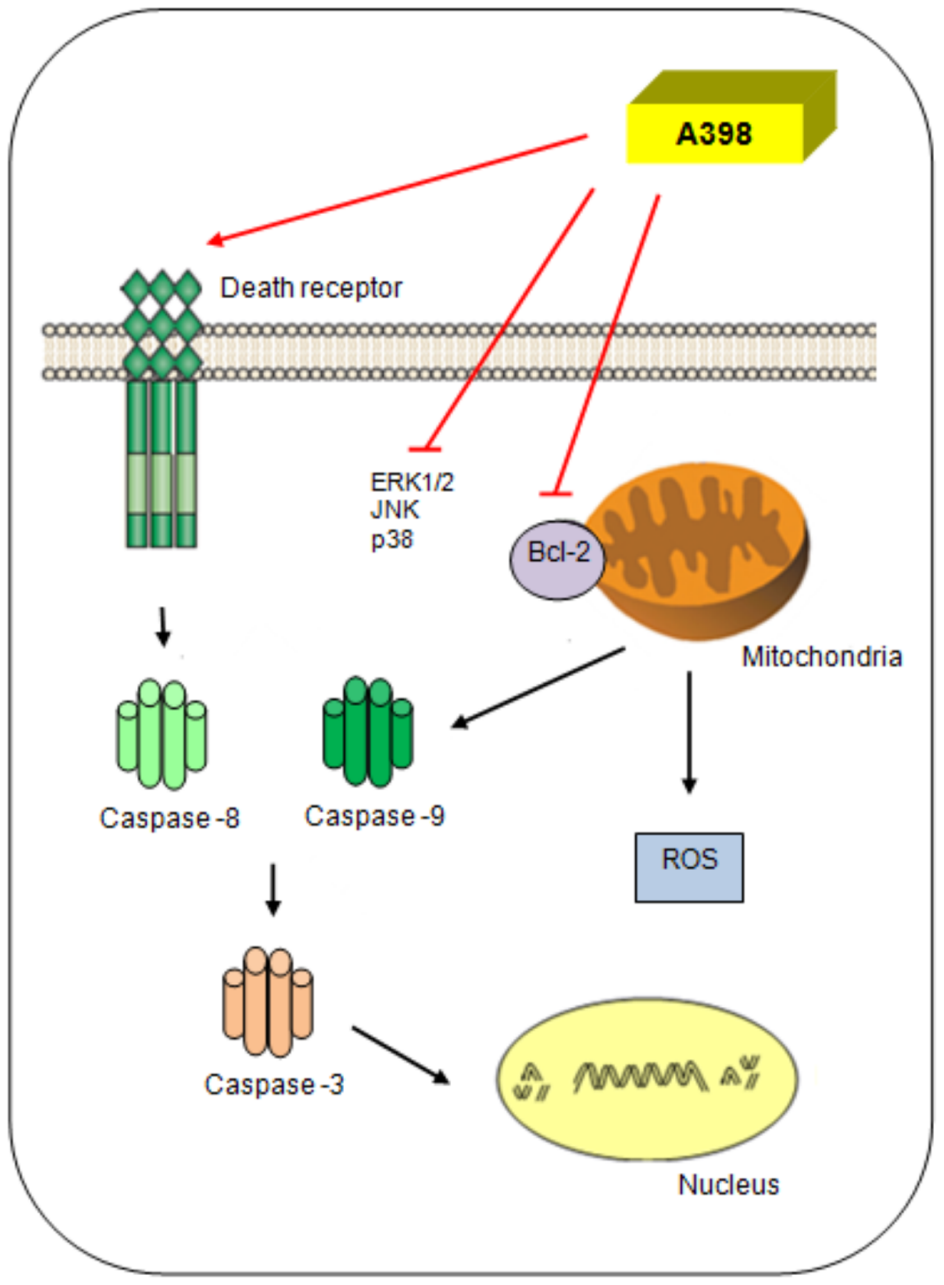
Hypothesized scheme of A398 events. For how compound A398 induced apoptosis in the leukemic HL-60 cells through the activation of the extrinsic and intrinsic pathways of death and its action on molecular targets, such as caspases, the anti-apoptotic protein Bcl-2 and MAPK proteins (ERK1/2, JNK and p38).

Studies indicate that an antitumor agent, in order to act effectively and more selectively in the treatment of cancer and to overcome the problem of drug resistance, should act in various molecular targets involved in tumorigenesis and the acquisition of the phenotype of multiple resistance to drugs [38], [31]. In this scenario, A398 showed low cytotoxicity against normal cells and was cytotoxic to several human tumor cell lines, inducing apoptosis in the leukemic HL-60 cells through the activation of the extrinsic and intrinsic pathways of death and acting on molecular targets that are amenable to therapeutic intervention, such as caspases, the anti-apoptotic protein Bcl-2 and MAPKs (as indicated in the graphical abstract, Figure 5). Taken together, the current study suggested that A398 has an anticancer potential, proving to be a good candidate for preclinical research. Further studies *in vivo* are necessary to evaluate drug efficacy in animal models and clarify the detailed mechanism(s) involved in the observed antitumor effect.

## Supporting Information

File S1 **Combined file of supporting figures. Figure S1. Induction of apoptosis in HL-60 lineage.** Cells were treated with derivative A398 (4, 6 and 8 µM) or with Etoposide (5 µM) for 1 h (A), 3 h (B) or 6 h (C). The cells were stained with annexin V-alexa fluor 488 and PI and evaluated by flow cytometry. The dual parametric dot plots show nonapoptotic live cells in the lower left quadrant (annexin V^−^/PI^−^), early apoptotic cells in the lower right quadrant (annexin V^+^/PI^−^), late apoptotic cells in upper right quadrant (annexin V^+^/PI^+^) or necrotic cells in the upper left (annexin V^−^/PI^+^). **Figure S2. Modification of mitochondrial transmembrane potential (ΔΨm) in HL-60 lineage.** Cells were treated with derivative A398 (4, 6 and 8 µM) or with etoposide (5 µM) for 1 h (A), 3 h (B) or 6 h (C). The cells were stained with TMRM and the percentage of depolarized cells was quantified by flow cytometry. Right indicator: % cells with ΔΨm normal. Left indicator: % depolarized cells. CCCP (50 µM) was included as a positive control. **Figure S3. Analysis of cell cycle and DNA fragmentation in HL-60 lineage.** Cells were treated with derivative A398 (2, 4 and 6 µM) or with etoposide (5 µM) for 1 h (A), 3 h (B) or 6 h (C). After treatment, cells were stained with PI and analyzed by flow cytometry. Indicator 1: % cells in sub-G_1_. Indicator 2: % cells in G_1_ phase. Indicator 3: % cells in S phase. Indicator 4: % cells in G_2_/M phases. **Figure S4. Induction of ROS generation in HL-60 lineage.** Cells were treated with derivative A398 (4, 6 and 8 µM) or etoposide (5 µM) for 1 h (A), 3 h (B) or 6 h (C). They were then labeled with H_2_- DCFH-DA and ROS production was quantified by flow cytometry. Indicator on the right: % cells with increased levels of ROS. H_2_O_2_ (50 µM) was used as a positive control.
(ZIP)
