# Supplementary material for: Compound A398, a Novel Podophyllotoxin Analogue: Cytotoxicity and Induction of Apoptosis in Human Leukemia Cells
Source: PLoS One. 2014 Sep 15;9(9):e107404. doi: 10.1371/journal.pone.0107404 (PMC4164611; doi:10.1371/journal.pone.0107404)
Supplement: File S1 — Combined file of supporting figures. Figure S1. Induction of apoptosis in HL-60 lineage. Cells were treated with derivative A398 (4, 6 and 8 µM) or with Etoposide (5 µM) for 1 h (A), 3 h (B) or 6 h (C). The cells were stained with annexin V-alexa fluor 488 and PI and evaluated by flow cytometry. The dual parametric dot plots show nonapoptotic live cells in the lower left quadrant (annexin V−/PI−), early apoptotic cells in the lower right quadrant (annexin V+/PI−), late apoptotic cells in upper right quadrant (annexin V+/PI+) or necrotic cells in the upper left (annexin V−/PI+). Figure S2. Modification of mitochondrial transmembrane potential (ΔΨm) in HL-60 lineage. Cells were treated with derivative A398 (4, 6 and 8 µM) or with etoposide (5 µM) for 1 h (A), 3 h (B) or 6 h (C). The cells were stained with TMRM and the percentage of depolarized cells was quantified by flow cytometry. Right indicator: % cells with ΔΨm normal. Left indicator: % depolarized cells. CCCP (50 µM) was included as a positive control. Figure S3. Analysis of cell cycle and DNA fragmentation in HL-60 lineage. Cells were treated with derivative A398 (2, 4 and 6 µM) or with etoposide (5 µM) for 1 h (A), 3 h (B) or 6 h (C). After treatment, cells were stained with PI and analyzed by flow cytometry. Indicator 1: % cells in sub-G1. Indicator 2: % cells in G1 phase. Indicator 3: % cells in S phase. Indicator 4: % cells in G2/M phases. Figure S4. Induction of ROS generation in HL-60 lineage. Cells were treated with derivative A398 (4, 6 and 8 µM) or etoposide (5 µM) for 1 h (A), 3 h (B) or 6 h (C). They were then labeled with H2- DCFH-DA and ROS production was quantified by flow cytometry. Indicator on the right: % cells with increased levels of ROS. H2O2 (50 µM) was used as a positive control. (ZIP) [file pone.0107404.s001.zip › LEGEND_Figs_Suppl.pdf]

LEGEND, SUPPLEMENTARY FIGURES

Figure S1. Induction of apoptosis in HL-60 lineage. Cells were treated with derivative A398 (4, 6 and 8 µM) or with Etoposide (5 µM) for 1h (A), 3h (B) or 6h (C). The cells were stained with annexin V-alexa fluor 488 and PI and evaluated by flow cytometry. The dual parametric dot plots show nonapoptotic live cells in the lower left quadrant (annexin V^-/PI^-), early apoptotic cells in the lower right quadrant (annexin V^+/PI^-), late apoptotic cells in upper right quadrant (annexin V^+/PI^+) or necrotic cells in the upper left (annexin V^-/PI^+).

Figure S2. Modification of mitochondrial transmembrane potential (ΔΨm) in HL-60 lineage. Cells were treated with derivative A398 (4, 6 and 8 µM) or with etoposide (5 µM) for 1h (A), 3h (B) or 6h (C). The cells were stained with TMRM and the percentage of depolarized cells was quantified by flow cytometry. Right indicator: % cells with ΔΨm normal. Left indicator: % depolarized cells. CCCP (50 µM) was included as a positive control.

Figure S3. Analysis of cell cycle and DNA fragmentation in HL-60 lineage. Cells were treated with derivative A398 (2, 4 and 6 µM) or with etoposide (5 µM) for 1h (A), 3h (B) or 6h (C). After treatment, cells were stained with PI and analyzed by flow cytometry. Indicator 1: % cells in sub-G_1. Indicator 2: % cells in G_1 phase. Indicator 3: % cells in S phase. Indicator 4: % cells in G_2/M phases.

Figure S4. Induction of ROS generation in HL-60 lineage. Cells were treated with derivative A398 (4, 6 and 8 µM) or etoposide (5 µM) for 1h (A), 3h (B) or 6h (C). They were then labeled with H_2- DCFH-DA and ROS production was quantified by flow cytometry. Indicator on the right: % cells with increased levels of ROS. H_2O_2 (50 µM) was used as a positive control.
