# Supplementary figures and images for: Compound A398, a Novel Podophyllotoxin Analogue: Cytotoxicity and Induction of Apoptosis in Human Leukemia Cells
Source: PLoS One. 2014 Sep 15;9(9):e107404. doi: 10.1371/journal.pone.0107404 (PMC4164611; doi:10.1371/journal.pone.0107404)

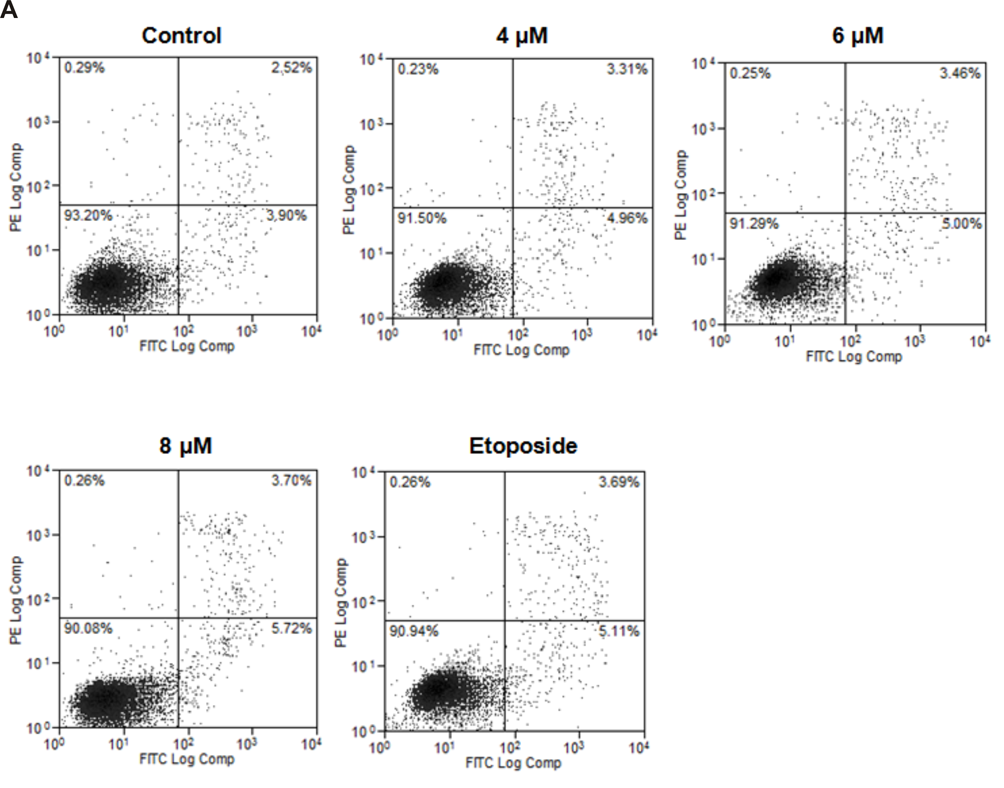

Supplement: File S1 — Combined file of supporting figures. Figure S1. Induction of apoptosis in HL-60 lineage. Cells were treated with derivative A398 (4, 6 and 8 µM) or with Etoposide (5 µM) for 1 h (A), 3 h (B) or 6 h (C). The cells were stained with annexin V-alexa fluor 488 and PI and evaluated by flow cytometry. The dual parametric dot plots show nonapoptotic live cells in the lower left quadrant (annexin V−/PI−), early apoptotic cells in the lower right quadrant (annexin V+/PI−), late apoptotic cells in upper right quadrant (annexin V+/PI+) or necrotic cells in the upper left (annexin V−/PI+). Figure S2. Modification of mitochondrial transmembrane potential (ΔΨm) in HL-60 lineage. Cells were treated with derivative A398 (4, 6 and 8 µM) or with etoposide (5 µM) for 1 h (A), 3 h (B) or 6 h (C). The cells were stained with TMRM and the percentage of depolarized cells was quantified by flow cytometry. Right indicator: % cells with ΔΨm normal. Left indicator: % depolarized cells. CCCP (50 µM) was included as a positive control. Figure S3. Analysis of cell cycle and DNA fragmentation in HL-60 lineage. Cells were treated with derivative A398 (2, 4 and 6 µM) or with etoposide (5 µM) for 1 h (A), 3 h (B) or 6 h (C). After treatment, cells were stained with PI and analyzed by flow cytometry. Indicator 1: % cells in sub-G1. Indicator 2: % cells in G1 phase. Indicator 3: % cells in S phase. Indicator 4: % cells in G2/M phases. Figure S4. Induction of ROS generation in HL-60 lineage. Cells were treated with derivative A398 (4, 6 and 8 µM) or etoposide (5 µM) for 1 h (A), 3 h (B) or 6 h (C). They were then labeled with H2- DCFH-DA and ROS production was quantified by flow cytometry. Indicator on the right: % cells with increased levels of ROS. H2O2 (50 µM) was used as a positive control. (ZIP) [file pone.0107404.s001.zip › Sup-Fig1A.tif]

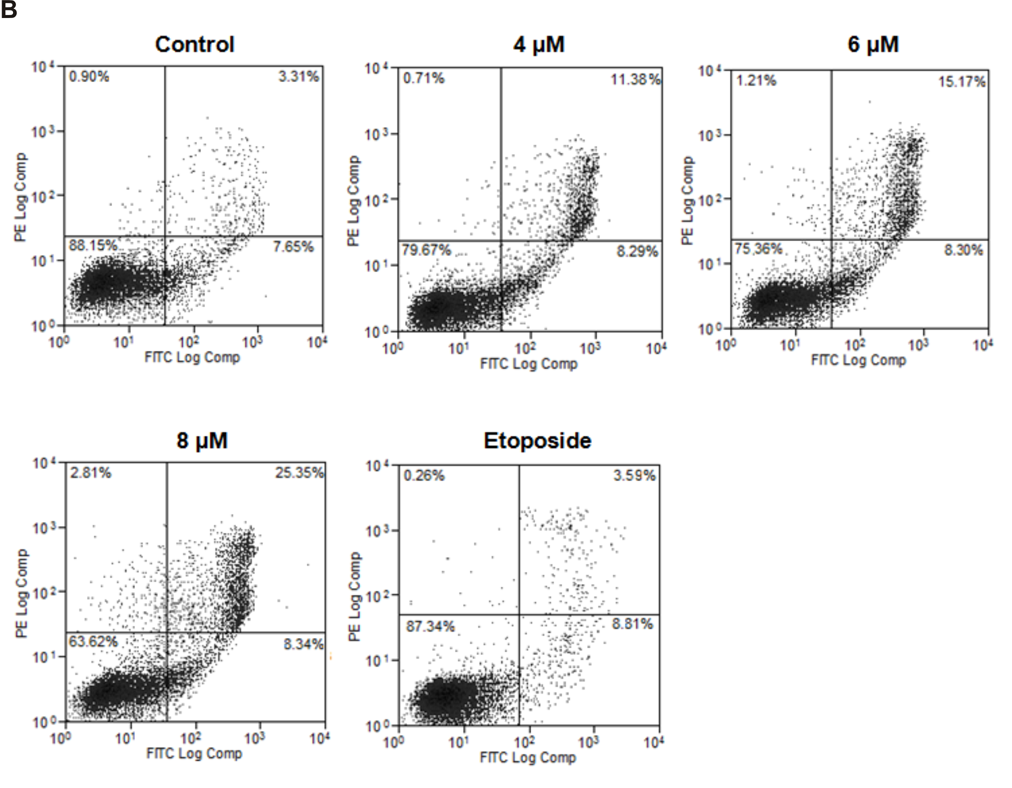

Supplement: File S1 — Combined file of supporting figures. Figure S1. Induction of apoptosis in HL-60 lineage. Cells were treated with derivative A398 (4, 6 and 8 µM) or with Etoposide (5 µM) for 1 h (A), 3 h (B) or 6 h (C). The cells were stained with annexin V-alexa fluor 488 and PI and evaluated by flow cytometry. The dual parametric dot plots show nonapoptotic live cells in the lower left quadrant (annexin V−/PI−), early apoptotic cells in the lower right quadrant (annexin V+/PI−), late apoptotic cells in upper right quadrant (annexin V+/PI+) or necrotic cells in the upper left (annexin V−/PI+). Figure S2. Modification of mitochondrial transmembrane potential (ΔΨm) in HL-60 lineage. Cells were treated with derivative A398 (4, 6 and 8 µM) or with etoposide (5 µM) for 1 h (A), 3 h (B) or 6 h (C). The cells were stained with TMRM and the percentage of depolarized cells was quantified by flow cytometry. Right indicator: % cells with ΔΨm normal. Left indicator: % depolarized cells. CCCP (50 µM) was included as a positive control. Figure S3. Analysis of cell cycle and DNA fragmentation in HL-60 lineage. Cells were treated with derivative A398 (2, 4 and 6 µM) or with etoposide (5 µM) for 1 h (A), 3 h (B) or 6 h (C). After treatment, cells were stained with PI and analyzed by flow cytometry. Indicator 1: % cells in sub-G1. Indicator 2: % cells in G1 phase. Indicator 3: % cells in S phase. Indicator 4: % cells in G2/M phases. Figure S4. Induction of ROS generation in HL-60 lineage. Cells were treated with derivative A398 (4, 6 and 8 µM) or etoposide (5 µM) for 1 h (A), 3 h (B) or 6 h (C). They were then labeled with H2- DCFH-DA and ROS production was quantified by flow cytometry. Indicator on the right: % cells with increased levels of ROS. H2O2 (50 µM) was used as a positive control. (ZIP) [file pone.0107404.s001.zip › Sup-Fig1B.tif]

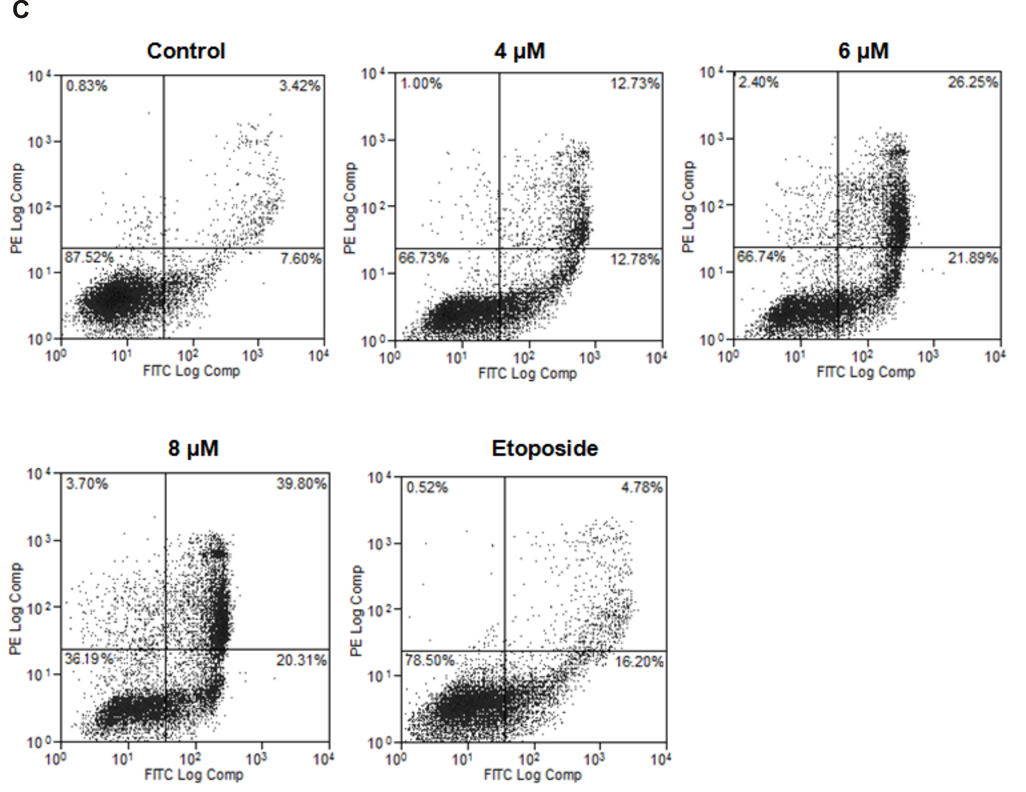

Supplement: File S1 — Combined file of supporting figures. Figure S1. Induction of apoptosis in HL-60 lineage. Cells were treated with derivative A398 (4, 6 and 8 µM) or with Etoposide (5 µM) for 1 h (A), 3 h (B) or 6 h (C). The cells were stained with annexin V-alexa fluor 488 and PI and evaluated by flow cytometry. The dual parametric dot plots show nonapoptotic live cells in the lower left quadrant (annexin V−/PI−), early apoptotic cells in the lower right quadrant (annexin V+/PI−), late apoptotic cells in upper right quadrant (annexin V+/PI+) or necrotic cells in the upper left (annexin V−/PI+). Figure S2. Modification of mitochondrial transmembrane potential (ΔΨm) in HL-60 lineage. Cells were treated with derivative A398 (4, 6 and 8 µM) or with etoposide (5 µM) for 1 h (A), 3 h (B) or 6 h (C). The cells were stained with TMRM and the percentage of depolarized cells was quantified by flow cytometry. Right indicator: % cells with ΔΨm normal. Left indicator: % depolarized cells. CCCP (50 µM) was included as a positive control. Figure S3. Analysis of cell cycle and DNA fragmentation in HL-60 lineage. Cells were treated with derivative A398 (2, 4 and 6 µM) or with etoposide (5 µM) for 1 h (A), 3 h (B) or 6 h (C). After treatment, cells were stained with PI and analyzed by flow cytometry. Indicator 1: % cells in sub-G1. Indicator 2: % cells in G1 phase. Indicator 3: % cells in S phase. Indicator 4: % cells in G2/M phases. Figure S4. Induction of ROS generation in HL-60 lineage. Cells were treated with derivative A398 (4, 6 and 8 µM) or etoposide (5 µM) for 1 h (A), 3 h (B) or 6 h (C). They were then labeled with H2- DCFH-DA and ROS production was quantified by flow cytometry. Indicator on the right: % cells with increased levels of ROS. H2O2 (50 µM) was used as a positive control. (ZIP) [file pone.0107404.s001.zip › Sup-Fig1C.tif]

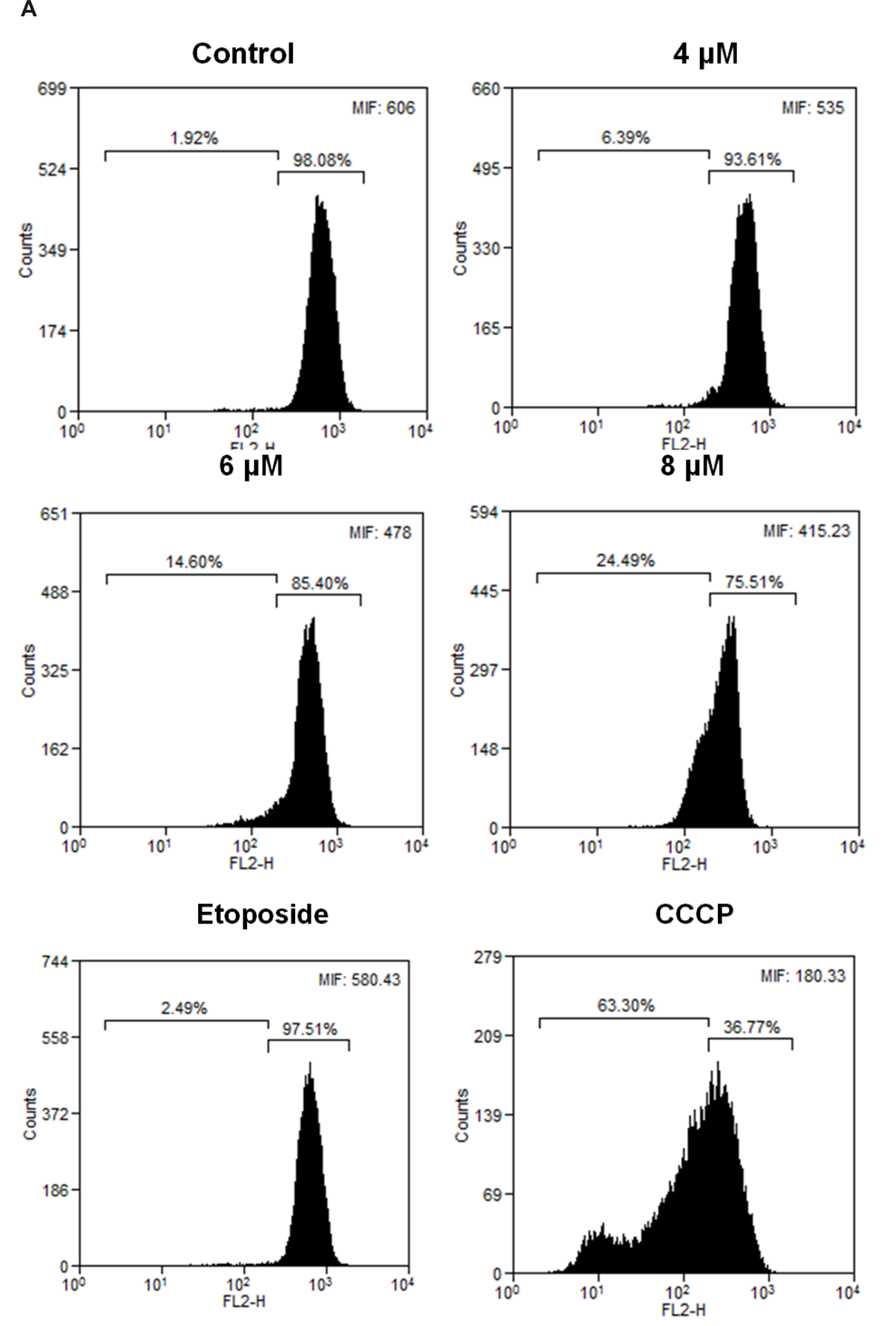

Supplement: File S1 — Combined file of supporting figures. Figure S1. Induction of apoptosis in HL-60 lineage. Cells were treated with derivative A398 (4, 6 and 8 µM) or with Etoposide (5 µM) for 1 h (A), 3 h (B) or 6 h (C). The cells were stained with annexin V-alexa fluor 488 and PI and evaluated by flow cytometry. The dual parametric dot plots show nonapoptotic live cells in the lower left quadrant (annexin V−/PI−), early apoptotic cells in the lower right quadrant (annexin V+/PI−), late apoptotic cells in upper right quadrant (annexin V+/PI+) or necrotic cells in the upper left (annexin V−/PI+). Figure S2. Modification of mitochondrial transmembrane potential (ΔΨm) in HL-60 lineage. Cells were treated with derivative A398 (4, 6 and 8 µM) or with etoposide (5 µM) for 1 h (A), 3 h (B) or 6 h (C). The cells were stained with TMRM and the percentage of depolarized cells was quantified by flow cytometry. Right indicator: % cells with ΔΨm normal. Left indicator: % depolarized cells. CCCP (50 µM) was included as a positive control. Figure S3. Analysis of cell cycle and DNA fragmentation in HL-60 lineage. Cells were treated with derivative A398 (2, 4 and 6 µM) or with etoposide (5 µM) for 1 h (A), 3 h (B) or 6 h (C). After treatment, cells were stained with PI and analyzed by flow cytometry. Indicator 1: % cells in sub-G1. Indicator 2: % cells in G1 phase. Indicator 3: % cells in S phase. Indicator 4: % cells in G2/M phases. Figure S4. Induction of ROS generation in HL-60 lineage. Cells were treated with derivative A398 (4, 6 and 8 µM) or etoposide (5 µM) for 1 h (A), 3 h (B) or 6 h (C). They were then labeled with H2- DCFH-DA and ROS production was quantified by flow cytometry. Indicator on the right: % cells with increased levels of ROS. H2O2 (50 µM) was used as a positive control. (ZIP) [file pone.0107404.s001.zip › Sup-Fig2-A.tif]

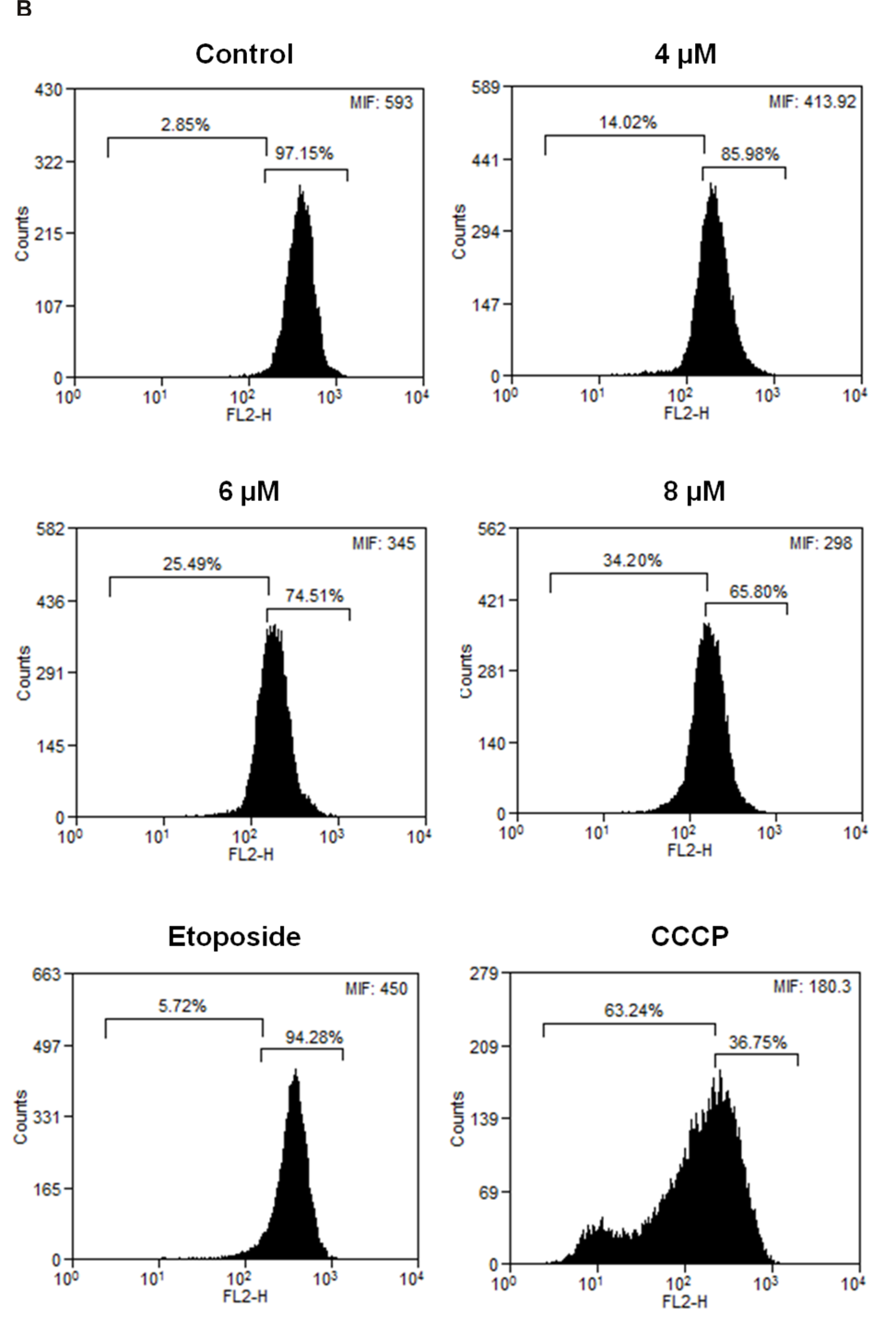

Supplement: File S1 — Combined file of supporting figures. Figure S1. Induction of apoptosis in HL-60 lineage. Cells were treated with derivative A398 (4, 6 and 8 µM) or with Etoposide (5 µM) for 1 h (A), 3 h (B) or 6 h (C). The cells were stained with annexin V-alexa fluor 488 and PI and evaluated by flow cytometry. The dual parametric dot plots show nonapoptotic live cells in the lower left quadrant (annexin V−/PI−), early apoptotic cells in the lower right quadrant (annexin V+/PI−), late apoptotic cells in upper right quadrant (annexin V+/PI+) or necrotic cells in the upper left (annexin V−/PI+). Figure S2. Modification of mitochondrial transmembrane potential (ΔΨm) in HL-60 lineage. Cells were treated with derivative A398 (4, 6 and 8 µM) or with etoposide (5 µM) for 1 h (A), 3 h (B) or 6 h (C). The cells were stained with TMRM and the percentage of depolarized cells was quantified by flow cytometry. Right indicator: % cells with ΔΨm normal. Left indicator: % depolarized cells. CCCP (50 µM) was included as a positive control. Figure S3. Analysis of cell cycle and DNA fragmentation in HL-60 lineage. Cells were treated with derivative A398 (2, 4 and 6 µM) or with etoposide (5 µM) for 1 h (A), 3 h (B) or 6 h (C). After treatment, cells were stained with PI and analyzed by flow cytometry. Indicator 1: % cells in sub-G1. Indicator 2: % cells in G1 phase. Indicator 3: % cells in S phase. Indicator 4: % cells in G2/M phases. Figure S4. Induction of ROS generation in HL-60 lineage. Cells were treated with derivative A398 (4, 6 and 8 µM) or etoposide (5 µM) for 1 h (A), 3 h (B) or 6 h (C). They were then labeled with H2- DCFH-DA and ROS production was quantified by flow cytometry. Indicator on the right: % cells with increased levels of ROS. H2O2 (50 µM) was used as a positive control. (ZIP) [file pone.0107404.s001.zip › Sup-Fig2-B.tif]

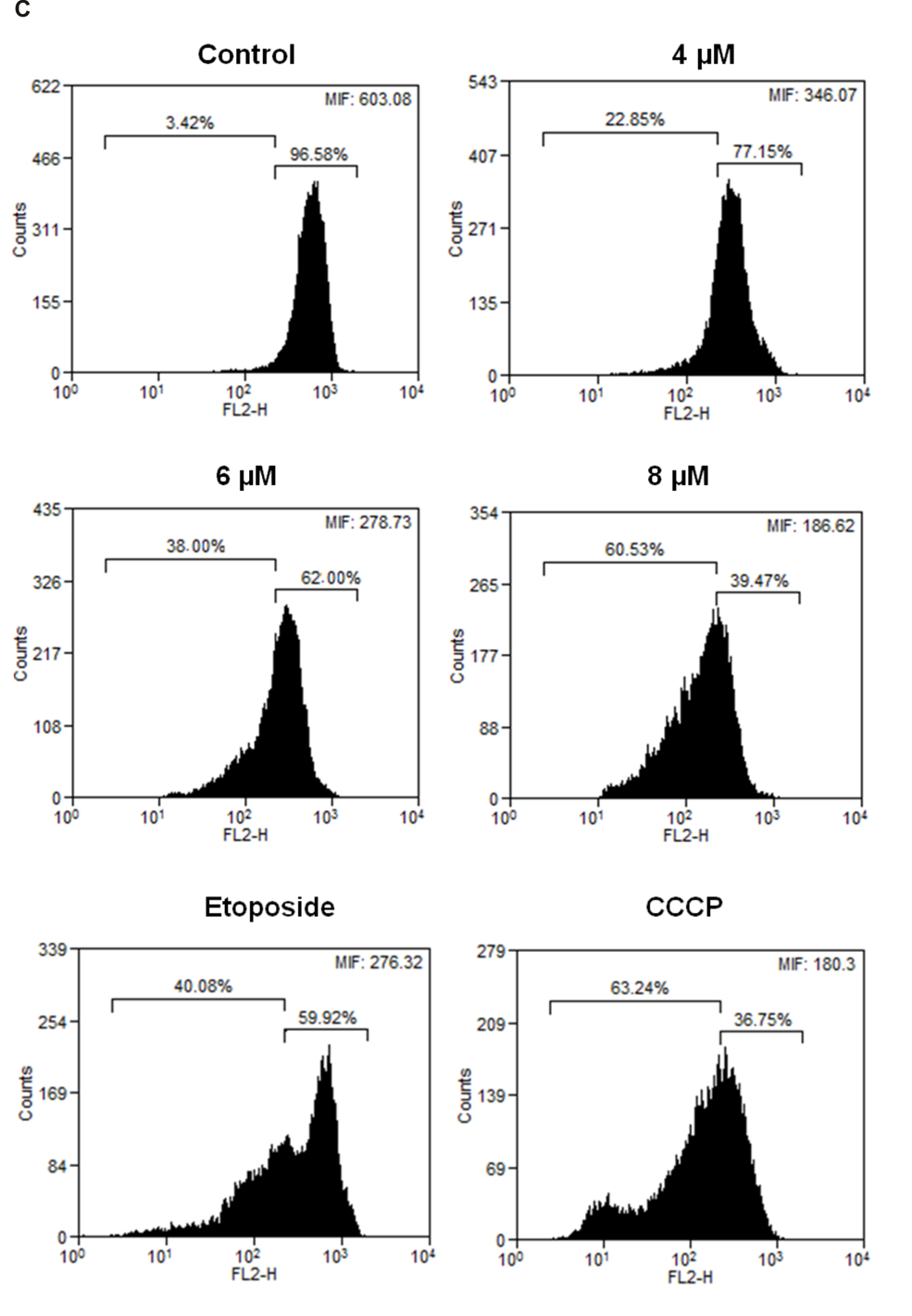

Supplement: File S1 — Combined file of supporting figures. Figure S1. Induction of apoptosis in HL-60 lineage. Cells were treated with derivative A398 (4, 6 and 8 µM) or with Etoposide (5 µM) for 1 h (A), 3 h (B) or 6 h (C). The cells were stained with annexin V-alexa fluor 488 and PI and evaluated by flow cytometry. The dual parametric dot plots show nonapoptotic live cells in the lower left quadrant (annexin V−/PI−), early apoptotic cells in the lower right quadrant (annexin V+/PI−), late apoptotic cells in upper right quadrant (annexin V+/PI+) or necrotic cells in the upper left (annexin V−/PI+). Figure S2. Modification of mitochondrial transmembrane potential (ΔΨm) in HL-60 lineage. Cells were treated with derivative A398 (4, 6 and 8 µM) or with etoposide (5 µM) for 1 h (A), 3 h (B) or 6 h (C). The cells were stained with TMRM and the percentage of depolarized cells was quantified by flow cytometry. Right indicator: % cells with ΔΨm normal. Left indicator: % depolarized cells. CCCP (50 µM) was included as a positive control. Figure S3. Analysis of cell cycle and DNA fragmentation in HL-60 lineage. Cells were treated with derivative A398 (2, 4 and 6 µM) or with etoposide (5 µM) for 1 h (A), 3 h (B) or 6 h (C). After treatment, cells were stained with PI and analyzed by flow cytometry. Indicator 1: % cells in sub-G1. Indicator 2: % cells in G1 phase. Indicator 3: % cells in S phase. Indicator 4: % cells in G2/M phases. Figure S4. Induction of ROS generation in HL-60 lineage. Cells were treated with derivative A398 (4, 6 and 8 µM) or etoposide (5 µM) for 1 h (A), 3 h (B) or 6 h (C). They were then labeled with H2- DCFH-DA and ROS production was quantified by flow cytometry. Indicator on the right: % cells with increased levels of ROS. H2O2 (50 µM) was used as a positive control. (ZIP) [file pone.0107404.s001.zip › Sup-Fig2-C.tif]

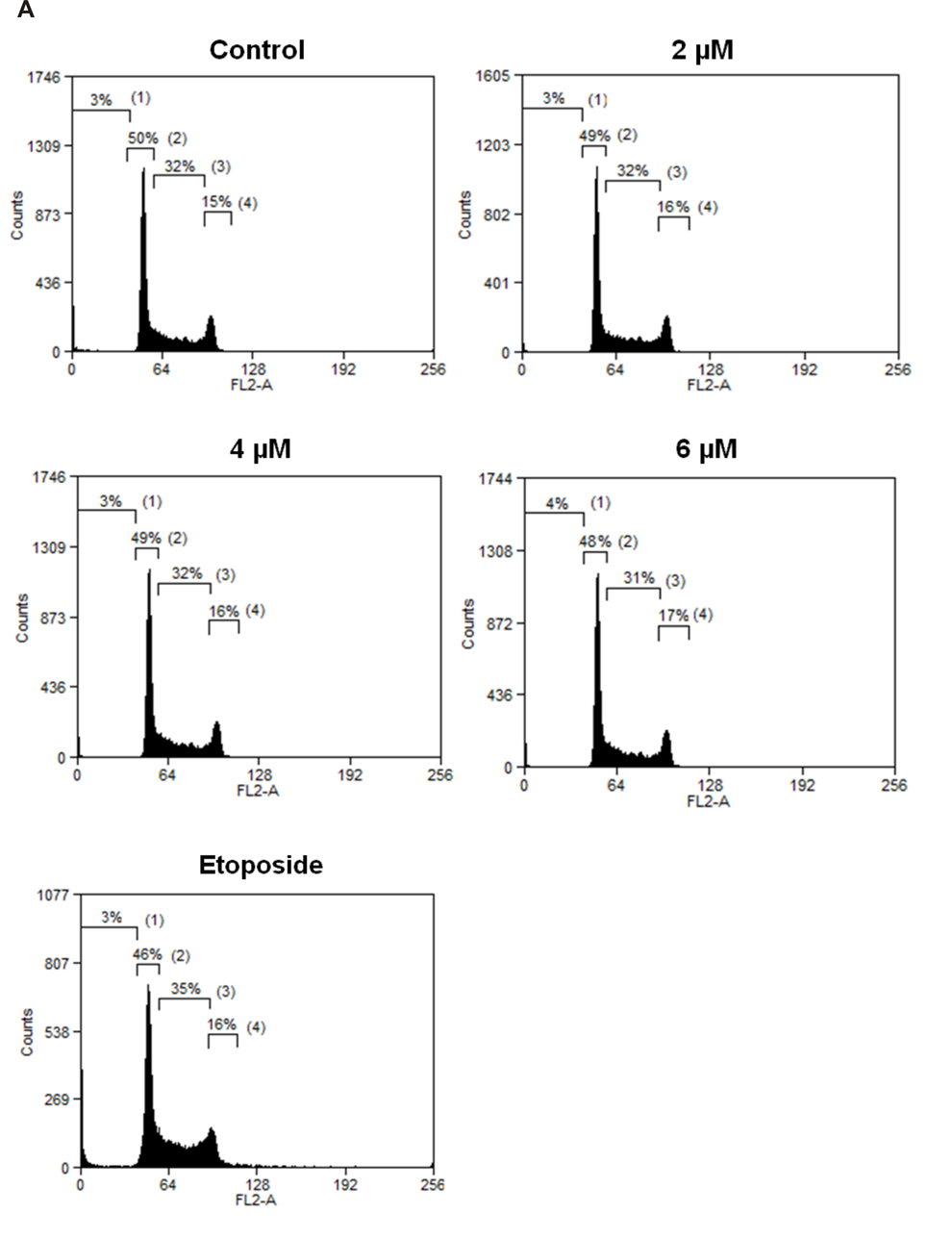

Supplement: File S1 — Combined file of supporting figures. Figure S1. Induction of apoptosis in HL-60 lineage. Cells were treated with derivative A398 (4, 6 and 8 µM) or with Etoposide (5 µM) for 1 h (A), 3 h (B) or 6 h (C). The cells were stained with annexin V-alexa fluor 488 and PI and evaluated by flow cytometry. The dual parametric dot plots show nonapoptotic live cells in the lower left quadrant (annexin V−/PI−), early apoptotic cells in the lower right quadrant (annexin V+/PI−), late apoptotic cells in upper right quadrant (annexin V+/PI+) or necrotic cells in the upper left (annexin V−/PI+). Figure S2. Modification of mitochondrial transmembrane potential (ΔΨm) in HL-60 lineage. Cells were treated with derivative A398 (4, 6 and 8 µM) or with etoposide (5 µM) for 1 h (A), 3 h (B) or 6 h (C). The cells were stained with TMRM and the percentage of depolarized cells was quantified by flow cytometry. Right indicator: % cells with ΔΨm normal. Left indicator: % depolarized cells. CCCP (50 µM) was included as a positive control. Figure S3. Analysis of cell cycle and DNA fragmentation in HL-60 lineage. Cells were treated with derivative A398 (2, 4 and 6 µM) or with etoposide (5 µM) for 1 h (A), 3 h (B) or 6 h (C). After treatment, cells were stained with PI and analyzed by flow cytometry. Indicator 1: % cells in sub-G1. Indicator 2: % cells in G1 phase. Indicator 3: % cells in S phase. Indicator 4: % cells in G2/M phases. Figure S4. Induction of ROS generation in HL-60 lineage. Cells were treated with derivative A398 (4, 6 and 8 µM) or etoposide (5 µM) for 1 h (A), 3 h (B) or 6 h (C). They were then labeled with H2- DCFH-DA and ROS production was quantified by flow cytometry. Indicator on the right: % cells with increased levels of ROS. H2O2 (50 µM) was used as a positive control. (ZIP) [file pone.0107404.s001.zip › Sup-Fig3-A.tif]

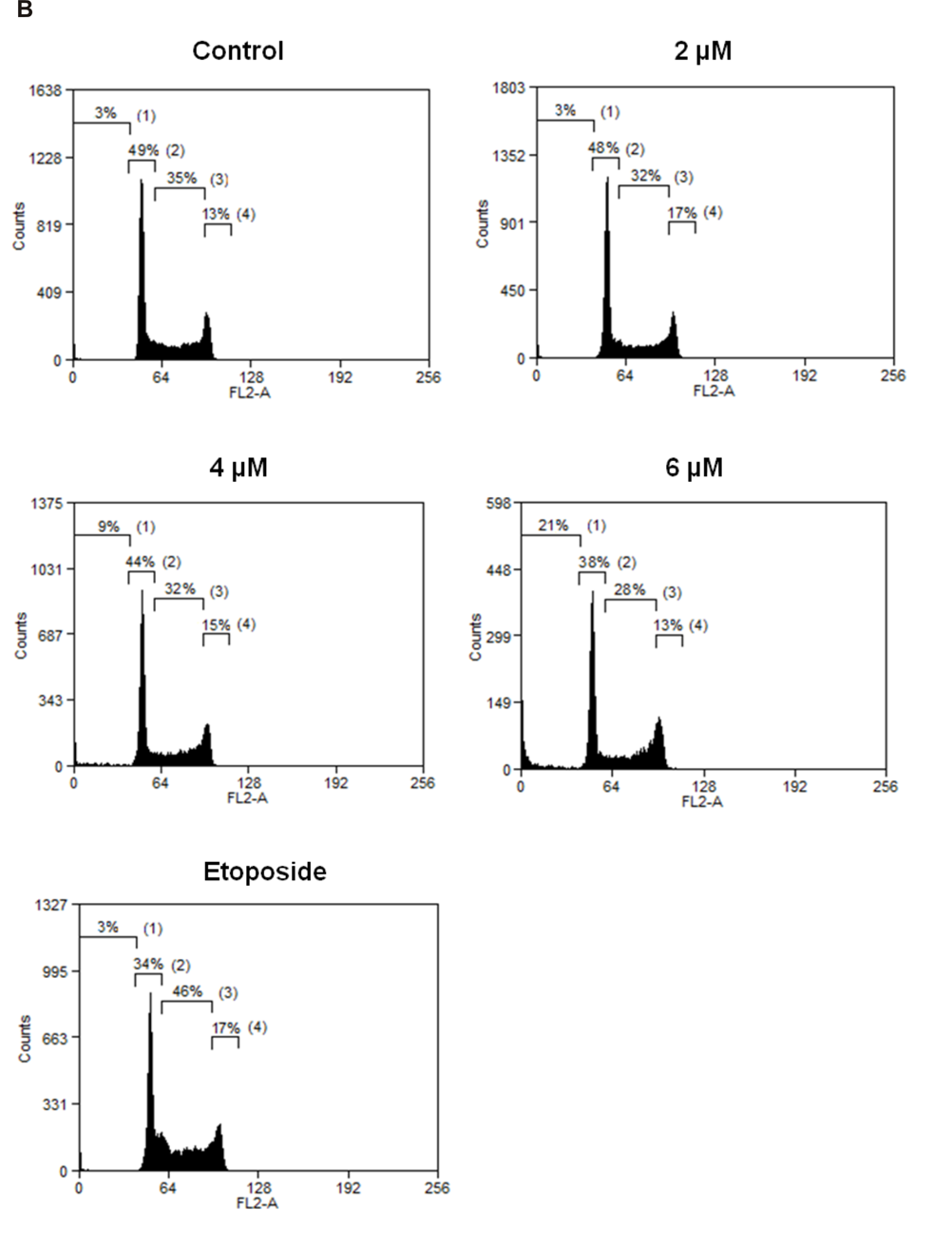

Supplement: File S1 — Combined file of supporting figures. Figure S1. Induction of apoptosis in HL-60 lineage. Cells were treated with derivative A398 (4, 6 and 8 µM) or with Etoposide (5 µM) for 1 h (A), 3 h (B) or 6 h (C). The cells were stained with annexin V-alexa fluor 488 and PI and evaluated by flow cytometry. The dual parametric dot plots show nonapoptotic live cells in the lower left quadrant (annexin V−/PI−), early apoptotic cells in the lower right quadrant (annexin V+/PI−), late apoptotic cells in upper right quadrant (annexin V+/PI+) or necrotic cells in the upper left (annexin V−/PI+). Figure S2. Modification of mitochondrial transmembrane potential (ΔΨm) in HL-60 lineage. Cells were treated with derivative A398 (4, 6 and 8 µM) or with etoposide (5 µM) for 1 h (A), 3 h (B) or 6 h (C). The cells were stained with TMRM and the percentage of depolarized cells was quantified by flow cytometry. Right indicator: % cells with ΔΨm normal. Left indicator: % depolarized cells. CCCP (50 µM) was included as a positive control. Figure S3. Analysis of cell cycle and DNA fragmentation in HL-60 lineage. Cells were treated with derivative A398 (2, 4 and 6 µM) or with etoposide (5 µM) for 1 h (A), 3 h (B) or 6 h (C). After treatment, cells were stained with PI and analyzed by flow cytometry. Indicator 1: % cells in sub-G1. Indicator 2: % cells in G1 phase. Indicator 3: % cells in S phase. Indicator 4: % cells in G2/M phases. Figure S4. Induction of ROS generation in HL-60 lineage. Cells were treated with derivative A398 (4, 6 and 8 µM) or etoposide (5 µM) for 1 h (A), 3 h (B) or 6 h (C). They were then labeled with H2- DCFH-DA and ROS production was quantified by flow cytometry. Indicator on the right: % cells with increased levels of ROS. H2O2 (50 µM) was used as a positive control. (ZIP) [file pone.0107404.s001.zip › Sup-Fig3-B.tif]

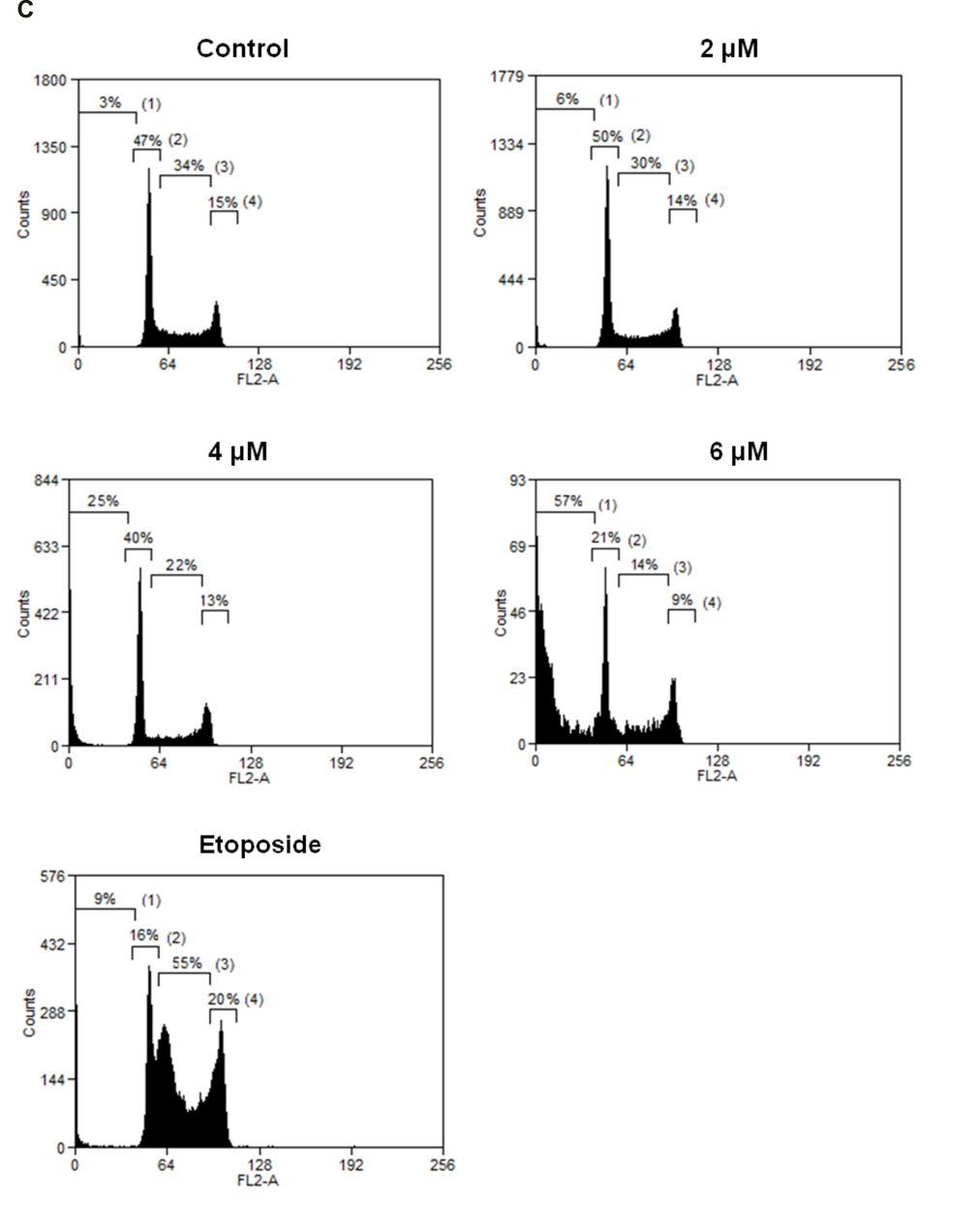

Supplement: File S1 — Combined file of supporting figures. Figure S1. Induction of apoptosis in HL-60 lineage. Cells were treated with derivative A398 (4, 6 and 8 µM) or with Etoposide (5 µM) for 1 h (A), 3 h (B) or 6 h (C). The cells were stained with annexin V-alexa fluor 488 and PI and evaluated by flow cytometry. The dual parametric dot plots show nonapoptotic live cells in the lower left quadrant (annexin V−/PI−), early apoptotic cells in the lower right quadrant (annexin V+/PI−), late apoptotic cells in upper right quadrant (annexin V+/PI+) or necrotic cells in the upper left (annexin V−/PI+). Figure S2. Modification of mitochondrial transmembrane potential (ΔΨm) in HL-60 lineage. Cells were treated with derivative A398 (4, 6 and 8 µM) or with etoposide (5 µM) for 1 h (A), 3 h (B) or 6 h (C). The cells were stained with TMRM and the percentage of depolarized cells was quantified by flow cytometry. Right indicator: % cells with ΔΨm normal. Left indicator: % depolarized cells. CCCP (50 µM) was included as a positive control. Figure S3. Analysis of cell cycle and DNA fragmentation in HL-60 lineage. Cells were treated with derivative A398 (2, 4 and 6 µM) or with etoposide (5 µM) for 1 h (A), 3 h (B) or 6 h (C). After treatment, cells were stained with PI and analyzed by flow cytometry. Indicator 1: % cells in sub-G1. Indicator 2: % cells in G1 phase. Indicator 3: % cells in S phase. Indicator 4: % cells in G2/M phases. Figure S4. Induction of ROS generation in HL-60 lineage. Cells were treated with derivative A398 (4, 6 and 8 µM) or etoposide (5 µM) for 1 h (A), 3 h (B) or 6 h (C). They were then labeled with H2- DCFH-DA and ROS production was quantified by flow cytometry. Indicator on the right: % cells with increased levels of ROS. H2O2 (50 µM) was used as a positive control. (ZIP) [file pone.0107404.s001.zip › Sup-Fig3-C.tif]

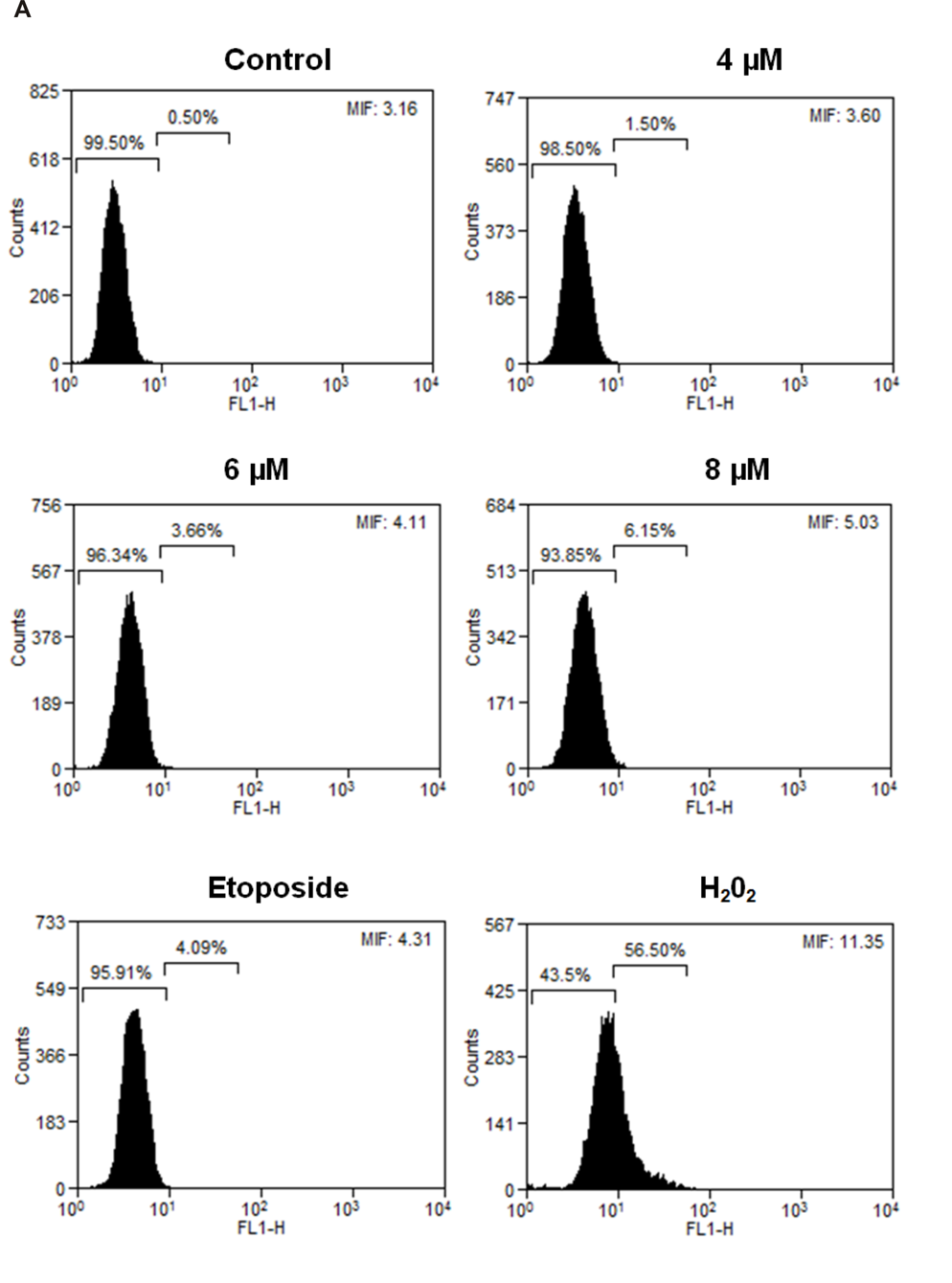

Supplement: File S1 — Combined file of supporting figures. Figure S1. Induction of apoptosis in HL-60 lineage. Cells were treated with derivative A398 (4, 6 and 8 µM) or with Etoposide (5 µM) for 1 h (A), 3 h (B) or 6 h (C). The cells were stained with annexin V-alexa fluor 488 and PI and evaluated by flow cytometry. The dual parametric dot plots show nonapoptotic live cells in the lower left quadrant (annexin V−/PI−), early apoptotic cells in the lower right quadrant (annexin V+/PI−), late apoptotic cells in upper right quadrant (annexin V+/PI+) or necrotic cells in the upper left (annexin V−/PI+). Figure S2. Modification of mitochondrial transmembrane potential (ΔΨm) in HL-60 lineage. Cells were treated with derivative A398 (4, 6 and 8 µM) or with etoposide (5 µM) for 1 h (A), 3 h (B) or 6 h (C). The cells were stained with TMRM and the percentage of depolarized cells was quantified by flow cytometry. Right indicator: % cells with ΔΨm normal. Left indicator: % depolarized cells. CCCP (50 µM) was included as a positive control. Figure S3. Analysis of cell cycle and DNA fragmentation in HL-60 lineage. Cells were treated with derivative A398 (2, 4 and 6 µM) or with etoposide (5 µM) for 1 h (A), 3 h (B) or 6 h (C). After treatment, cells were stained with PI and analyzed by flow cytometry. Indicator 1: % cells in sub-G1. Indicator 2: % cells in G1 phase. Indicator 3: % cells in S phase. Indicator 4: % cells in G2/M phases. Figure S4. Induction of ROS generation in HL-60 lineage. Cells were treated with derivative A398 (4, 6 and 8 µM) or etoposide (5 µM) for 1 h (A), 3 h (B) or 6 h (C). They were then labeled with H2- DCFH-DA and ROS production was quantified by flow cytometry. Indicator on the right: % cells with increased levels of ROS. H2O2 (50 µM) was used as a positive control. (ZIP) [file pone.0107404.s001.zip › Sup-Fig4-A.tif]

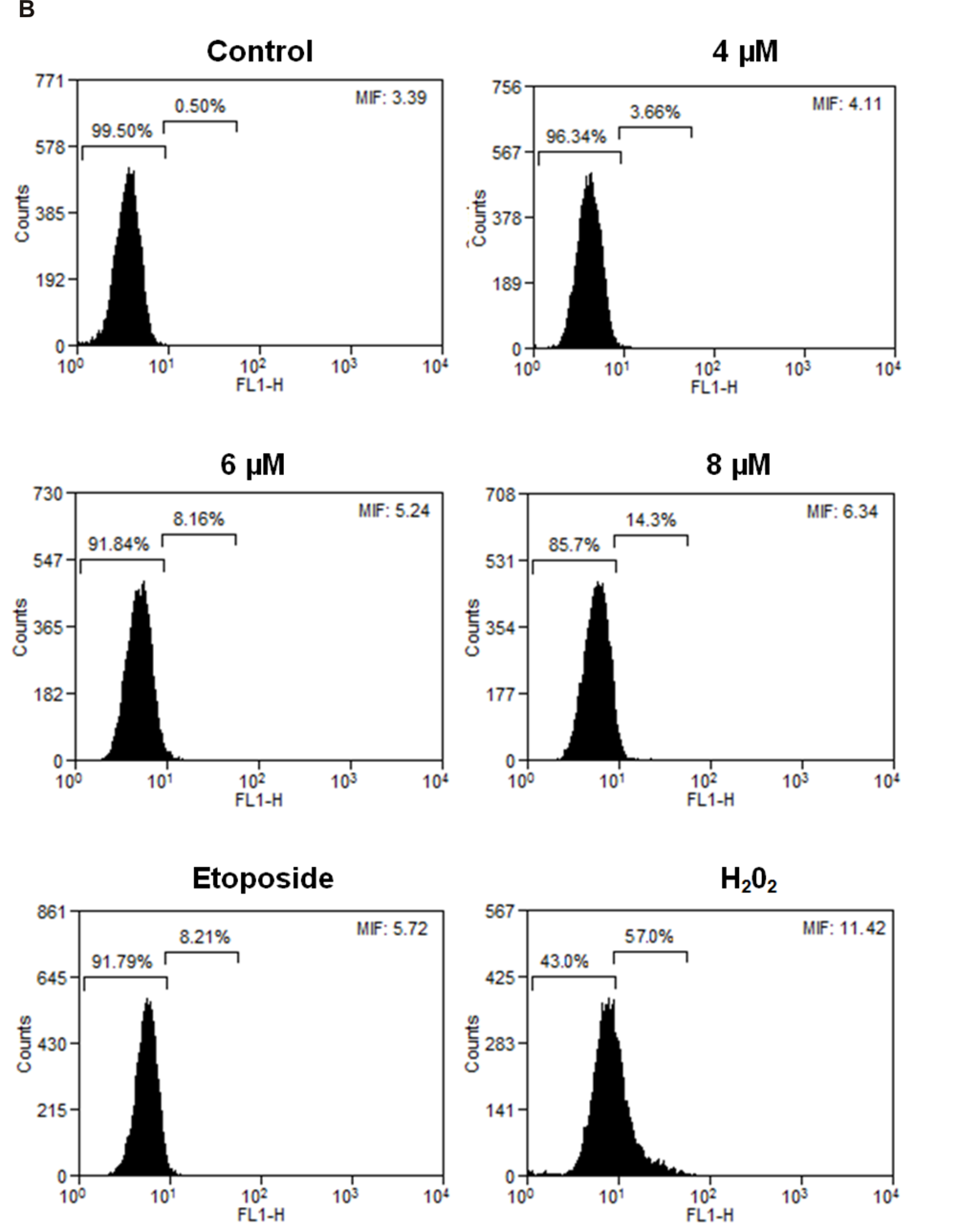

Supplement: File S1 — Combined file of supporting figures. Figure S1. Induction of apoptosis in HL-60 lineage. Cells were treated with derivative A398 (4, 6 and 8 µM) or with Etoposide (5 µM) for 1 h (A), 3 h (B) or 6 h (C). The cells were stained with annexin V-alexa fluor 488 and PI and evaluated by flow cytometry. The dual parametric dot plots show nonapoptotic live cells in the lower left quadrant (annexin V−/PI−), early apoptotic cells in the lower right quadrant (annexin V+/PI−), late apoptotic cells in upper right quadrant (annexin V+/PI+) or necrotic cells in the upper left (annexin V−/PI+). Figure S2. Modification of mitochondrial transmembrane potential (ΔΨm) in HL-60 lineage. Cells were treated with derivative A398 (4, 6 and 8 µM) or with etoposide (5 µM) for 1 h (A), 3 h (B) or 6 h (C). The cells were stained with TMRM and the percentage of depolarized cells was quantified by flow cytometry. Right indicator: % cells with ΔΨm normal. Left indicator: % depolarized cells. CCCP (50 µM) was included as a positive control. Figure S3. Analysis of cell cycle and DNA fragmentation in HL-60 lineage. Cells were treated with derivative A398 (2, 4 and 6 µM) or with etoposide (5 µM) for 1 h (A), 3 h (B) or 6 h (C). After treatment, cells were stained with PI and analyzed by flow cytometry. Indicator 1: % cells in sub-G1. Indicator 2: % cells in G1 phase. Indicator 3: % cells in S phase. Indicator 4: % cells in G2/M phases. Figure S4. Induction of ROS generation in HL-60 lineage. Cells were treated with derivative A398 (4, 6 and 8 µM) or etoposide (5 µM) for 1 h (A), 3 h (B) or 6 h (C). They were then labeled with H2- DCFH-DA and ROS production was quantified by flow cytometry. Indicator on the right: % cells with increased levels of ROS. H2O2 (50 µM) was used as a positive control. (ZIP) [file pone.0107404.s001.zip › Sup-Fig4-B.tif]

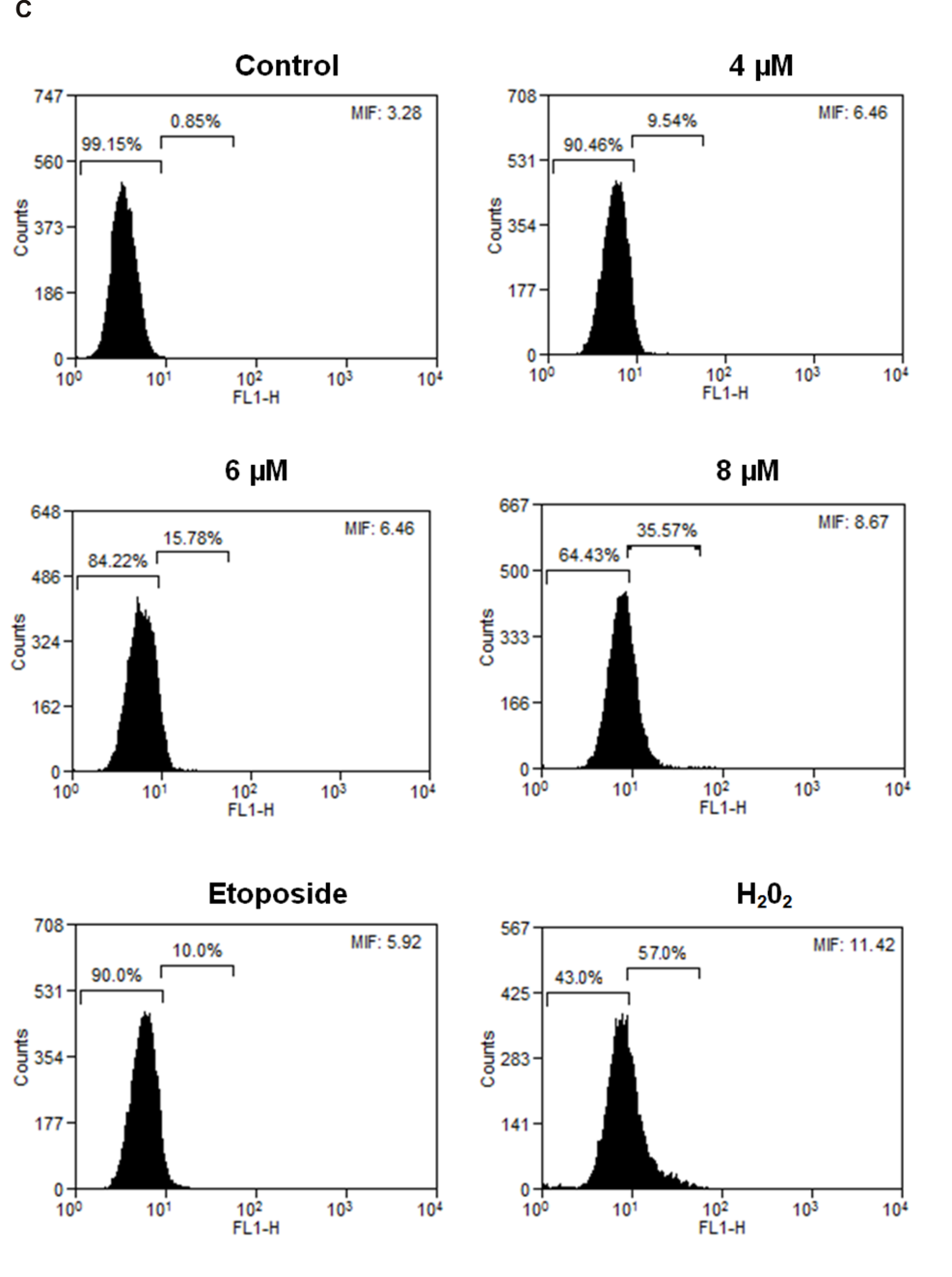

Supplement: File S1 — Combined file of supporting figures. Figure S1. Induction of apoptosis in HL-60 lineage. Cells were treated with derivative A398 (4, 6 and 8 µM) or with Etoposide (5 µM) for 1 h (A), 3 h (B) or 6 h (C). The cells were stained with annexin V-alexa fluor 488 and PI and evaluated by flow cytometry. The dual parametric dot plots show nonapoptotic live cells in the lower left quadrant (annexin V−/PI−), early apoptotic cells in the lower right quadrant (annexin V+/PI−), late apoptotic cells in upper right quadrant (annexin V+/PI+) or necrotic cells in the upper left (annexin V−/PI+). Figure S2. Modification of mitochondrial transmembrane potential (ΔΨm) in HL-60 lineage. Cells were treated with derivative A398 (4, 6 and 8 µM) or with etoposide (5 µM) for 1 h (A), 3 h (B) or 6 h (C). The cells were stained with TMRM and the percentage of depolarized cells was quantified by flow cytometry. Right indicator: % cells with ΔΨm normal. Left indicator: % depolarized cells. CCCP (50 µM) was included as a positive control. Figure S3. Analysis of cell cycle and DNA fragmentation in HL-60 lineage. Cells were treated with derivative A398 (2, 4 and 6 µM) or with etoposide (5 µM) for 1 h (A), 3 h (B) or 6 h (C). After treatment, cells were stained with PI and analyzed by flow cytometry. Indicator 1: % cells in sub-G1. Indicator 2: % cells in G1 phase. Indicator 3: % cells in S phase. Indicator 4: % cells in G2/M phases. Figure S4. Induction of ROS generation in HL-60 lineage. Cells were treated with derivative A398 (4, 6 and 8 µM) or etoposide (5 µM) for 1 h (A), 3 h (B) or 6 h (C). They were then labeled with H2- DCFH-DA and ROS production was quantified by flow cytometry. Indicator on the right: % cells with increased levels of ROS. H2O2 (50 µM) was used as a positive control. (ZIP) [file pone.0107404.s001.zip › Sup-Fig4-C.tif]
